# Down-regulation of BMAL1 by MiR-494-3p Promotes Hepatocellular Carcinoma Growth and Metastasis by Increasing GPAM-mediated Lipid Biosynthesis

**DOI:** 10.7150/ijbs.74951

**Published:** 2022-10-18

**Authors:** Yi Yang, Tao Yang, Zifeng Zhao, Hongxin Zhang, Peng Yuan, Gang Wang, Zheng Zhao, Jiaze An, Zhuomin Lyu, Jinliang Xing, Jibin Li

**Affiliations:** 1 Department of Pain Treatment, Tangdu Hospital, Fourth Military Medical University, Xi'an, Shaanxi, 710038, China.; 2 State Key Laboratory of Cancer Biology and Department of Physiology and Pathophysiology, Fourth Military Medical University, Xi'an, Shaanxi, 710032, China.; 3 Third Department of Medical Oncology, Shaanxi Provincial Cancer Hospital, Xi'an, Shaanxi, 710032, China.; 4 Department of Hepatobiliary Surgery, Xijing Hospital, Fourth Military Medical University, Xi'an, Shaanxi, 710032, China.; 5 Experimental Teaching Center of Basic Medicine, Fourth Military Medical University, Xi'an, Shaanxi, 710032, China.

**Keywords:** BMAL1, GPAM, EZH2, lipid biosynthesis, growth, metastasis, hepatocellular carcinoma

## Abstract

The circadian clock confers daily rhythmicity to many crucial biological processes and behaviors and its disruption is closely associated with carcinogenesis in several types of cancer. Brain and muscle arnt-like protein 1 (BMAL1) is a core circadian rhythm component in mammals and its dysregulation has been shown to contribute to aberrant metabolism in human diseases. However, the biological functions of BMAL1, especially its involvement in aberrant lipid metabolism in hepatocellular carcinoma (HCC), remain elusive. In the present study, we found that BMAL1 was frequently down-regulated in HCC cells mainly due to the up-regulation of miR-494-3p. Down-regulation of BMAL1 was significantly associated with poor survival in HCC patients. BMAL1 down-regulation promoted HCC cell growth and metastasis both *in vitro* and *in vivo*. Mechanistically, through cooperating with EZH2, BMAL1 transcriptionally suppressed the expression of glycerol-3-phosphate acyltransferase mitochondrial (GPAM), a key enzyme involved in the regulation of lipid biosynthesis, leading to reduced levels lysophosphatidic acid (LPA), which have long been known as mediator of oncogenesis. Particularly, treatment with SR8278, an activator of BMAL1, exhibited a therapeutic effect on HCC *in vitro* and *in vivo*. In conclusion, BMAL1 plays a critical anti-oncogenic role in HCC, providing strong research evidence for BMAL1 as a prospective target for HCC therapy.

## Introduction

The circadian clock is critical for the maintenance of daily rhythms of crucial biological processes and behaviors in almost all species, including prokaryotes and humans 1-3. In mammals, several clock genes maintain circadian rhythm by exerting effects on transcriptional and post-translational feedback loops 4, 5. Emerging evidence has shown a strong association between circadian dysrhythmia, for instance night shift work or irregular diet and diverse diseases, especially cancer 6. Circadian disruption has been causally linked to the development and progression of many human cancers, such as ovarian, breast, colorectal and gastric cancers 7. Considering the significant role of circadian disruption in the onset and development of these cancers, it is crucial to discern the mechanisms underlying circadian disruption-induced cancer onset and progression.

Brain and muscle arnt-like protein 1 (BMAL1) is a vital circadian rhythm component in mammals. It directly regulates the expression of circadian output genes and negative circadian loop genes by binding to the E-Box elements on the promoters of these genes 4, 8. Additionally, BMAL1 has been shown to play antitumor roles in many human cancers, such as colorectal cancer, head and neck squamous cell carcinoma, and ovarian cancer 9-12. In particular, accumulating evidence has shown that dysregulation of BMAL1 contributes to aberrant metabolism in various human diseases, such as liver insulin resistance, hyperlipidaemia and atherosclerosis 13-15. Nevertheless, the biological functions of BMAL1, especially its precise role in lipid metabolism reprogramming in cancer cells, remain elusive.

In this study, we analyzed the expression trend, clinical value and biological influences of BMAL1 in HCC. More significantly, the molecular mechanisms underlying BMAL1-induced HCC tumorigenesis were profoundly assessed.

## Materials and methods

### Public data set collection and Kaplan-Meier plotter

Public mRNA expression data from The Cancer Genome Atlas (TCGA) were selected for gene expression analyses. Kaplan-Meier plotter survival analysis platform was used for evaluation of the prognostic significance of BMAL1. Functional enrichment was accomplished via Kyoto encyclopedia of genes and genomes (KEGG) cascade assessment.

### Cell transfections

BMAL1 and GPAM overexpression vectors (50 nM), siRNAs against BMAL1 and GPAM (50 nM), miRNA-494-3p mimic (50 nM), anti-miRNA-494-3p (50 nM) and their respective negative controls were purchased from GenePharma Co, Ltd. (China). Then, they were transfected into HCC cells using Lipofectamine 2000 (Invitrogen, CA, USA), as instructed by the manufacturer. Sequences of siRNAs against BMAL1 and GPAM, as well as miRNA-494-3p mimic and anti-miRNA-494-3p are provided in Supplementary Table 1.

### Cell viability

The MTS cell viability (Promega Corporation, G3581) assay was performed to assess cell proliferation as per the manufacturers' instructions. First, 1000 HCC cells with different treatments were inoculated into 96-well-dish plates (020096, Xinyou Biotech, Hangzhou, China) and cultured for 12 h. Then, wells were supplemented with 20 μl MTS (0.2%)-PMS (0.092%; phenazine methosulfate, 20:1) solution and incubated for 2 h at 37 °C. Absorbance of each well was spectrophotometrically determined at 490 nm.

### Colony formation assay

For colony formation assessments, 1000 HCC cells with different treatments were inoculated into 6-well plates, incubated for 14 days, washed using PBS, fixed with 4% formaldehyde for 10 min at room temperature and stained by crystal violet. After washing twice in PBS, the number of colonies was determined.

### Ethynyldeoxyuridine (EdU) incorporation analysis

The Cell-Light EdU DNA Cell Proliferation Kit (RiboBio) was used in this assay as per the manufacturer's instructions. Briefly, the Edu solution was introduced into the cell culture medium at a concentration of 0.1%, followed by incubation for 2 h. Cells were collected, fixed using 4% formaldehyde and stained with staining mix for 20 min in the dark. Positive staining of EDU was visualized by fluorescent microscopy (DM5000B; Leica, Heerbrugg, Switzerland).

## Results

### BMAL1 is frequently down-regulated in HCC tissues and linked with tumor development and poor prognosis

**Figure 1:**
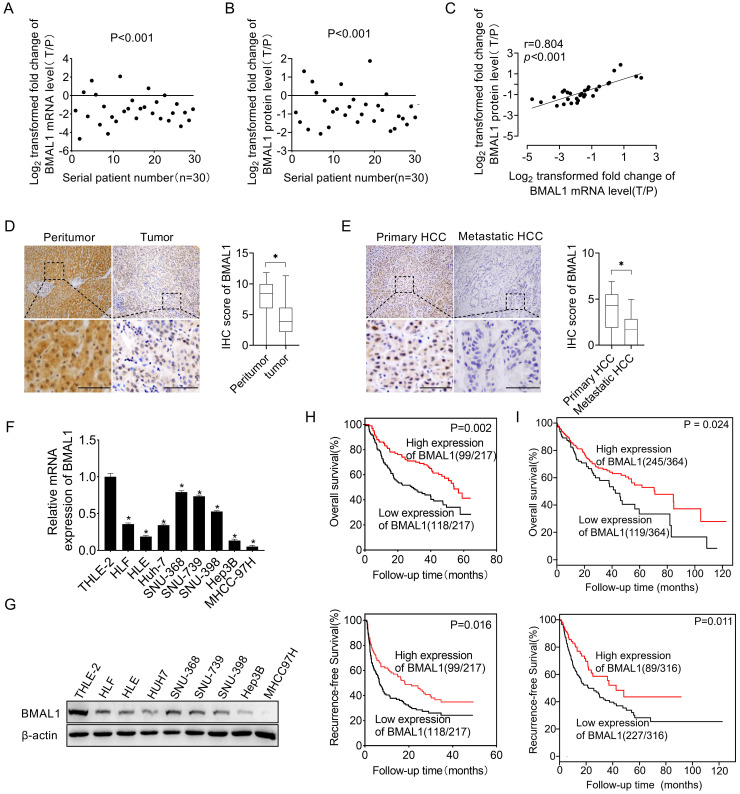
** Down-regulated BMAL1 in HCC tissues correlated with tumor progression and poor prognosis. (A-B)** qRT-PCR and Western blot analyses of BMAL1 in 30 paired tumor and peritumor tissues from HCC patients. Relative tumor to peritumor (T/P) expression ratio of BMAL1 was log2-transformed. qRT-PCR data are presented according to patient serial numbers, while patient serial numbers for Western blot data are rearranged as per expression levels. **(C)** Correlations between mRNA and protein levels of BMAL1 in 30 paired tissues from HCC patients. **(D)** Representative IHC-staining images (left), as well as IHC scores (right) for BMAL1 in 217 paired HCC tissues. **(E)** Representative IHC-staining images (left) and IHC scores (right) for BMAL1 in 36 paired primary and metastatic HCC tissues. *p < 0.05. Scale bar: 100 µm. **(F-G)** Expressions of BMAL1 in eight HCC cell lines and one normal human hepatocyte (HL-7702) were assessed by qRT-PCR and Western blot assays. **(H)** Kaplan-Meier assessments of overall (OS) and recurrence-free survival (RFS) outcomes for HCC patients based on BMAL1 expressions (n=217). **(I)** Bioinformatics analysis of OS and RFS outcomes for HCC patients as per the BMAL1 expression using the Kaplan-Meier plotter survival analysis platform (n=364). *p <0.05.

To investigate the function of BMAL1 in HCC, we first determined its expression level in 30 paired tumors, as well as in neighboring healthy tissues. qRT-PCR and Western blotting assays demonstrated that both mRNA and protein expression levels of BMAL1 were lower in tumor tissues than in paired neighboring healthy tissues (**Figure 1A, 1B and Figure S1**). In addition, we found a significant positive relationship between mRNA and protein levels of BMAL1 in individual patients (**Figure 1C**), suggesting that BMAL1 may be down-regulated at the transcriptional level. A similar pattern of BMAL1 expression was demonstrated through immunohistochemistry (IHC) analysis of a large dataset of 217 HCC tumor tissues. The results showed that BMAL1 expression level was significantly lower in HCC tissues compared with matching peritumor tissues (96.8%, 67.3% and 65.0% in fibrotic, cirrhotic and fibrotic+cirrhotic tissues, respectively) (**Figure 1D**). Further IHC analysis of 36 paired primary and metastatic HCC tissues showed that BMAL1 was markedly down-regulated in metastatic tumor tissues compared with primary HCC tissues (**Figure 1E**). Consistent with the data from HCC tissues, BMAL1 expression was also remarkably lower in eight HCC cell lines (HLE, HLF, Hep3B, SNU-368, SNU-398, SNU-739, Huh7 and MHCC97H) compared with that in normal hepatocyte THLE-2 (**Figure 1F and 1G**). We then evaluated the clinical significance of BMAL1 in HCC. As shown in **supplementary Table 3**, BMAL1 expression level was not associated with clinical and pathological features, such as Alpha Fetoprotein (AFP) level, age, Tumor Node Metastasis (TNM) stage, gender, and HBV infection. However, BMAL1 expression level was negatively associated with tumor size and distant metastasis. Kaplan-Meier assessment demonstrated that HCC patients with low BMAL1 expression level exhibited remarkably poor overall survival (OS) and relapse-free survival (RFS) compared with those with high BMAL1 expression (**Figure 1H**). This relationship was further validated by bioinformatics analysis based on Kaplan-Meier plotter survival analysis platform (**Figure 1I**). Furthermore, both univariate and multivariate Cox analysis revealed an association between higher BMAL1 expression and favorable survival outcomes, including OS (univariate HR: 0.544, 95% CI: 0.375-0.790; multivariate HR: 0.598, 95% CI: 0.403-0.889) and RFS (univariate HR: 0.504, 95% CI: 0.361-0.705; multivariate HR: 0.511, 95% CI: 0.358-0.731) in patients with HCC (**supplementary Table 4-7**). This suggests that BMAL1 is an independent prognostic factor in HCC. Collectively, these findings demonstrate that BMAL1 is frequently down-regulated in HCC cells, predicting poor survival for individuals with HCC.

### Silencing of *BMAL1* enhances the growth and metastasis of HCC cells

**Figure 2:**
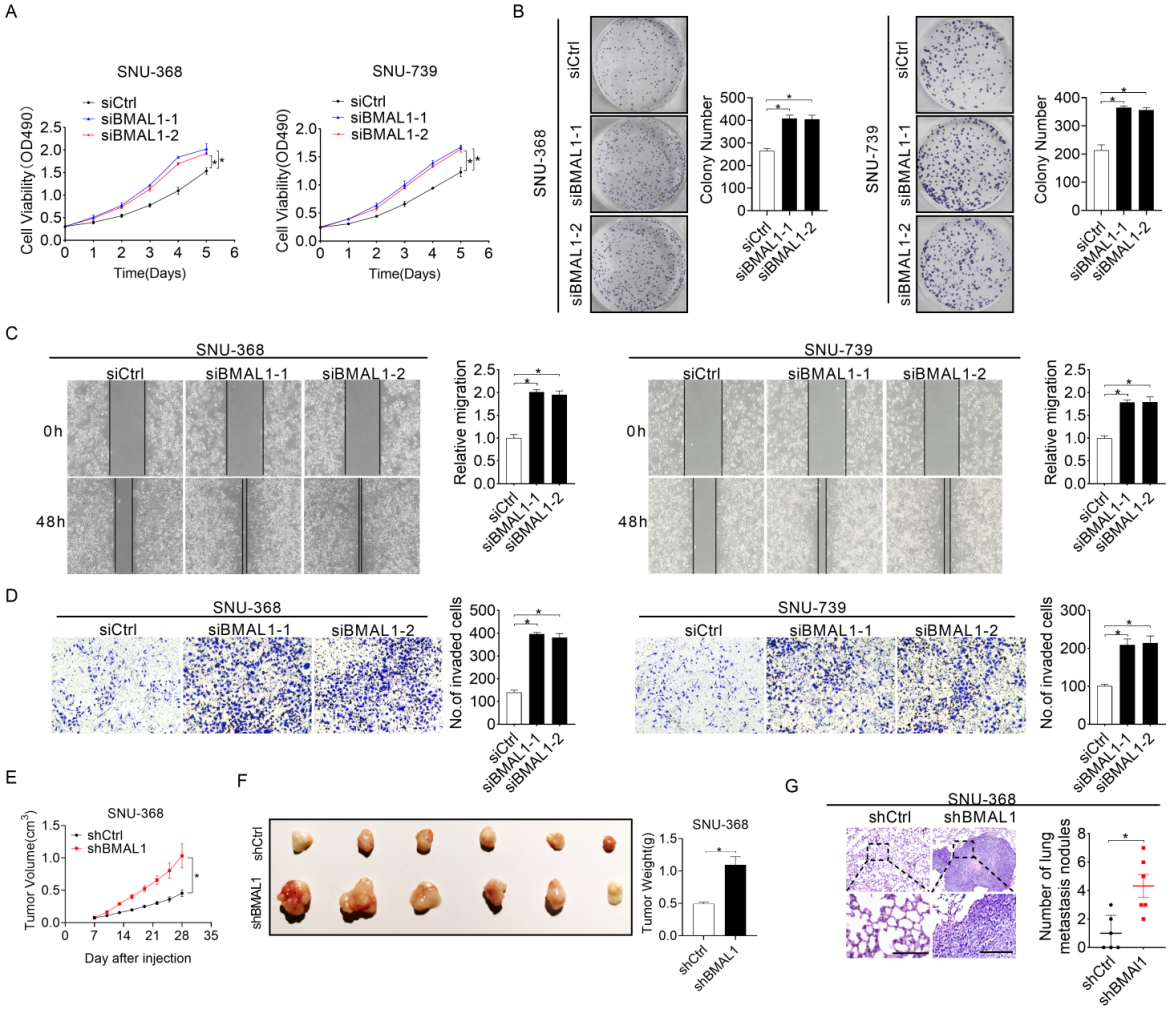
** Silencing of BMAL1 enhanced HCC cell proliferation and metastasis. (A)** MTS assay for SNU-368 and SNU-739 cells treated as specified (siBMAL1, siRNA against BMAL1; siCtrl, control siRNA). **(B)** Colony formation assay for SNU-368 and SNU-739 cells treated as specified. **(C)** Scratch-wound-healing analysis for migration abilities of SNU-368 and SNU-739 cells treated as specified. **(D)** Matrigel invasion assay for invasive abilities of SNU-368 and SNU-739 cells after treatments as specified. **(E) Subcutaneous** tumor-growth curves for HCC cells stably transfected with short-hairpin RNA targeting BMAL1 (n=6 mice/group). **(F)** Dissected subcutaneous tumors from sacrificed mice (left panel), with their weights (right panel) (n=6 mice/group). **(G)** Numbers of lung metastasis nodules in each group (n=6 mice/group). Scale bar, 100 µm. *p <0.05.

To systemically elucidate biological roles of BMAL1 in HCC cells, *BMAL1* was silenced in SNU-368 and SNU-739 cells as shown in **Figure 1F and 1G**. Silencing of *BMAL1* was validated with qRT-PCR and Western blotting assays (**Figure S2A and S2B**). MTS cell viability and colony formation evaluation demonstrated that SNU-368 and SNU-739 cells with *BMAL1* knockdown grew significantly faster than control cells (**Figure 2A-2B**). Considering the accelerated progression of the cell cycle and/or apoptosis resistance could contribute to increased cell proliferation, we evaluated the impact of *BMAL1* silencing on cell cycle distribution and apoptosis in SNU-368 and SNU-739 cells using flow cytometry. The number of SNU-368 and SNU-739 cells with *BMAL1* knockdown in G1 phase decreased, whereas those in S phase increased compared with control cells (**Figure S2D**). Congruently, EdU incorporation assessment demonstrated a higher proliferation of SNU-368 and SNU-739 cells with *BMAL1* knockdown compared with control cells (**Figure S2C**). To elucidate the mechanism by which BMAL1 suppresses G1-S cell cycle, the expression levels of key regulators of G1 cell cycle (CDK4, CDK6 and cyclin D1) were determined using Western blotting analysis. Our results showed that the expression levels of CDK4, cyclin D1 and CDK6 were higher in SNU-368 and SNU-739 cells with *BMAL1* silencing than in control cells (**Figure S2F**). Apoptosis analysis using flow cytometry indicated significantly lower proportion of apoptotic cells in SNU-368 and SNU-739 cells with *BMAL1* silencing compared with control cells (**Figure S2E**). This result was further validated by Western blotting analysis, showing that the secretion of cytochrome c from mitochondria to cytoplasm and cleavage of caspase 3, caspase 9 and PARP were remarkably decreased after *BMAL1* silencing in SNU-368 and SNU-739 cells (**Figure S2G**). Altogether, these results demonstrate that BMAL1 suppresses HCC growth by inhibiting G1-S cell cycle transition and increasing cell apoptosis. We then investigated the tumor-suppressive effect of BMAL1 on cell migration and infiltration. Scratch wound healing assessment showed that *BMAL1* silencing significantly enhanced the migration potential of SNU-368 and SNU-739 cells (**Figure 2C**). Matrigel invasion assessment also demonstrated that silencing of *BMAL1* promoted the invasion potential of SNU-368 and SNU-739 cells (**Figure 2D**). Considering that epithelial-mesenchymal transition (EMT) is a mechanism pivotal to tumor metastasis, we investigated the involvement of EMT in BMAL1-regulated HCC metastasis. As shown in **Figure S2H**, *BMAL1* silencing significantly promoted EMT, as evidenced by down-regulated epithelium regulators (E-cadherin and ZO-1) and up-regulated mesenchymal regulators (Vimentin and N-cadherin).

To further examine the suppressive role of BMAL1 in *in vivo* tumor growth, we established subcutaneous xenograft tumor models by administering SNU-368 cells harboring stable *BMAL1* knockdown or control cells into the left and right flanks of nude mice, respectively. As indicated in **Figure 2E**, tumor growth rate in the nude mice injected with *BMAL1* knockdown SUN-368 cells was remarkably faster than in those injected with control cells. Consistently, at the end of the evaluation, the average weight of tumors excised from the nude mice injected with *BMAL1* knockdown SUN-368 cells was also significantly higher than that of tumors from control mice (**Figure 2F**). Similar to the* in vitro* experiments, we found more proliferating cells and fewer apoptotic cells in *BMAL1* knockdown xenografts using Ki-67 staining and TUNEL assays, respectively (**Figures S2I and S2J**). In concordance with *in vitro* findings, *BMAL1* silencing also remarkably increased *in vivo* lung metastasis ability of SNU-368 cells (**Figure 2G**).

### Over-expression of BMAL1 attenuates HCC growth and metastasis

**Figure 3:**
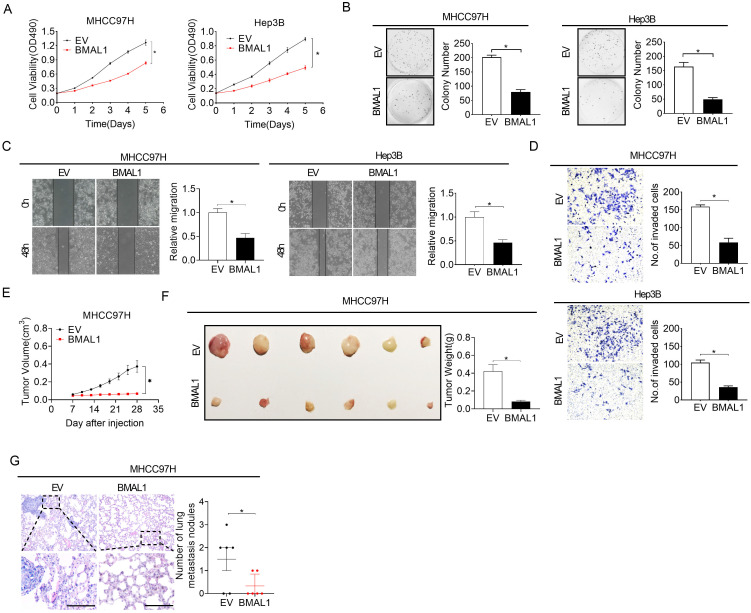
** Over-expressed BMAL1 attenuated HCC cell proliferation and metastasis. (A and B)** MTS and colony formation assays for determining the growth potential of MHCC97H and Hep-3B cells after treatment as indicated (BMAL1, expression vector encoding BMAL1; EV, empty vector). **(C and D)** Scratch-wound-healing and Matrigel invasion assays for assessment of metastatic abilities of MHCC97H and Hep-3B cells after treatments as indicated. **(E)** Subcutaneous tumor-growth curves for HCC cells stably transfected with the expression vector encoding BMAL1 (n=6 mice/group). **(F)** Dissected subcutaneous tumors (left panel) and their weights (right panel) (n=6 mice/group). **(G)** The number of lung metastasis nodules in each group (n=6 mice/group). Scale bar, 100 µm. *p <0.05.

To additionally confirm the suppressive role of BMAL1 in the growth and metastasis of HCC, we over-expressed BMAL1 by transfecting the BMAL1 expression vector or empty vector into MHCC97H and Hep3B cells with relatively low BMAL1 expression, as indicated in **Figure 1F and 1G**. The efficiency of BMAL1 over-expression was validated using qRT-PCR and Western blotting assays (**Figure S3A and S3B**). MTS cell viability and colony formation assessment showed a significantly lower cell viability and clonogenicity in MHCC97H and Hep3B cells over-expressing BMAL1 (**Figure 3A and 3B**). We also observed significantly lower proportions of proliferating cells and higher cell apoptosis when BMAL1 was over-expressed (**Figure S3C-S3E**). Western blotting analysis proved the mechanism underlying BMAL1-suppressed HCC growth and metastasis (**Figure S3F-S3G**). Scratch wound healing and transwell invasion assays further indicated that over-expression of BMAL1 distinctly repressed the migration and infiltration potential of MHCC97H and Hep3B cells (**Figure 3C and 3D**). Consistently, the EMT in MHCC97H and Hep3B cells was also suppressed when BMAL1 was over-expressed (**Figure S3H**). Furthermore, the *in vivo* tumor-suppressive effects of BMAL1 over-expression were also determined. As shown in **Figure 3E and 3F**, the forced expression of BMAL1 resulted in lower tumor growth rates and weights in MHCC97H cells compared with controls. Similar to the *in vitro* experiments, remarkably fewer proliferating cells and more apoptotic cells were found in xenografts overexpressing BMAL1 using Ki-67 staining and TUNEL assays, respectively (**Figure S3I and S3J**). Besides, the forced expression of BMAL1 significantly suppressed lung metastastic potential of MHCC97H cells (**Figure 3G**). Altogether, these results further confirmed the crucial anti-tumor role of BMAL1 in HCC.

### BMAL1 transcriptionally inhibits GPAM expression in an EZH2-dependent way in HCC cells

**Figure 4:**
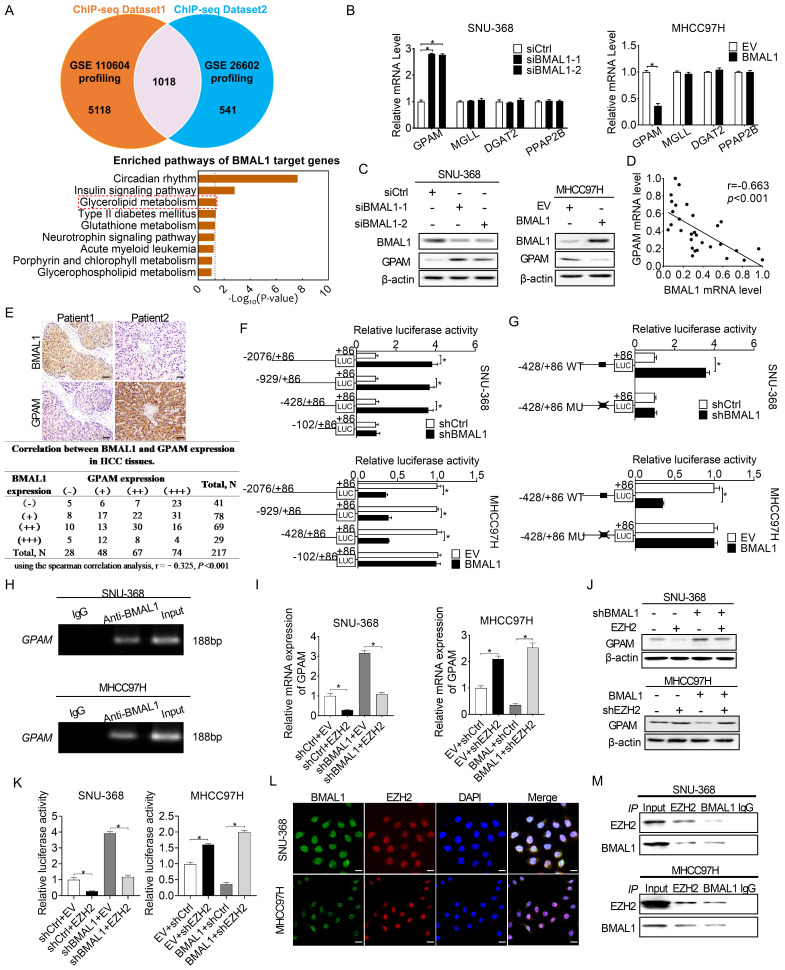
** BMAL1 transcriptionally repressed GPAM expressions in HCC cells in an EZH2-dependent manner. (A)** Potential transcriptional targets of BMAL1 in two BMAL1 chromatin immunoprecipitation (ChIP) sequencing datasets were analyzed. **(B and C)** qRT-PCR and Western blot assays for GPAM, MGLL, DGAT2 and PPAP2B expressions in SNU-368 as well as MHCC97H cells after specific treatments. **(D)** Scatter plot assessment of the relationship between mRNA expressions of BMAL1 and GPAM in tumor tissues from 30 HCC patients. **(E)** Representative immunohistochemical (IHC) staining results (upper panel) and the relationship between protein expressions (lower panel) of BMAL1 and GPAM in tumor tissues from in 217 HCC patients. Scale bar, 100 µm. **(F and G)** Relative luciferase activities of the GPAM promoter were determined in SNU-368 and MHCC97H cells after treatments as indicated. **(H)** Amplification of GPAM promoter sequence in ChIP DNA from SNU-368 and MHCC97H cells. Input and IgG were the positive and negative controls, respectively. **(I and J)** mRNA and protein levels of GPAM in SNU-368 and MHCC97H cells after specific treatments. **(K)** Relative luciferase activities of GPAM promoter in SNU-368 and MHCC97H cells after specific treatments. **(L)** Representative immunofluorescent images of BMAL1 and EZH2 in SNU-368 and MHCC97H cells. Scale bar, 25 µm. **(M)** Immunoprecipitation of BMAL1 and GPAM in SNU-368 and MHCC97H cells after specific treatments. *p <0.05.

BMAL1 has been established as the transcription factor that regulates the expression of several downstream genes 4. To determine the molecular mechanism underlying BMAL1 suppression of HCC cell growth and metastasis, we first analyzed potential transcriptional targets of BMAL1 using two public datasets of BMAL1 chromatin immunoprecipitation (ChIP) (GEO accession numbers: GSE26602 and GSE110604) 16, 17. A total of 1018 candidate transcriptional genes targeted by BMAL1 were found in both datasets (**Figure 4A**). Notably, these target genes of BMAL1 were enriched in glycerolipid metabolic pathway, which plays important roles in tumor progression (**Figure 4A**). Quantitative RT-PCR and Western blotting analysis revealed four genes (Glycerol-3-Phosphate Acyltransferase, *GPAM*; Monoglyceride Lipase, *MGLL*; Diacylglycerol O-Acyltransferase 2, *DGAT2*; and Phosphatidic acid phosphatase type 2B, *PPAP2B*) enriched in glycerolipid metabolic pathway. Among them, *GPAM* exhibited high expression when BMAL1 was silenced in SNU-368 cells and low expression when BMAL1 was over-expressed in MHCC97H cells (**Figure 4B and 4C**). Scatter plot analysis showed a negative relationship between mRNA expression levels of BMAL1 and GPAM in tumor tissues from 30 HCC individuals (**Figure 4D**). Similarly, a negative relationship between the protein expression levels of BMAL1 and GPAM was observed in 217 HCC tissues (**Figure 4E**) and 36-unpaired primary and metastasis tumor tissues from HCC patients (**Figure S4A and S4B**). Consistently, a negative relationship was also found in HCC and normal hepatic cell lines (**Figure S4C and S4D**). Moreover, the prognostic value of GPAM was assessed based on the IHC staining results. Kaplan-Meier survival curves indicated that HCC patients with high GPAM level have significantly worse survival outcomes compared with patients with low GPAM expression **(Figure S4E)**. To investigate whether BMAL1 down-regulated the expression of GPAM at transcriptional level, luciferase reporter analysis was applied. We found that constructs truncated from -2076 to -428 displayed similar BMAL1-linked transcriptional activities with only the construct from -428 to -102 completely alleviating the transcriptional activity of GPAM in all transfected cells (**Figure 4F**). These results were further supported by site-directed mutagenesis analysis, showing that the E-box (from -179 to -174) was critical to BMAL1-mediated suppression of GPAM expression (**Figure 4G**). ChIP-PCR assessment also demonstrated that BMAL1 docks directly to the promoter of GPAM (**Figure 4H**).

BMAL1 is a well-known transcription factor, which exerts its effects on the promoter region and transcriptionally activates downstream target genes 18. Nevertheless, we found that BMAL1 transcriptionally down-regulated GPAM expression in HCC cells. Given that EZH2 has been reported to be required for BMAL1 to suppress the transcription of several target genes 19, we postulated that EZH2 might be required for BMAL1 to down-regulate GPAM in HCC cells. To prove this relationship, we over-expressed EZH2 in SNU-368 cells harboring BMAL1 knockdown, and silenced EZH2 in MHCC97H cells with BMAL1 over-expression. As shown in **Figure 4I-4K** using qRT-PCR and Western blotting analysis, EZH2 overexpression significantly attenuated the impact of BMAL1 silencing on the expression of GPAM. In contrast, silencing of EZH2 alleviated the effect of BMAL1 on inhibiting the expression of GPAM. Additionally, double immunofluorescence staining and Co-IP assays supported the protein-protein interaction between BMAL1 and EZH2 in HCC cells (**Figure 4L and 4M**). Collectively, these results indicate that BMAL1 cooperates with EZH2 to suppress the transcription of GPAM in HCC cells.

### BMAL1 decreases glycerolipid synthesis in HCC by suppressing GPAM

**Figure 5:**
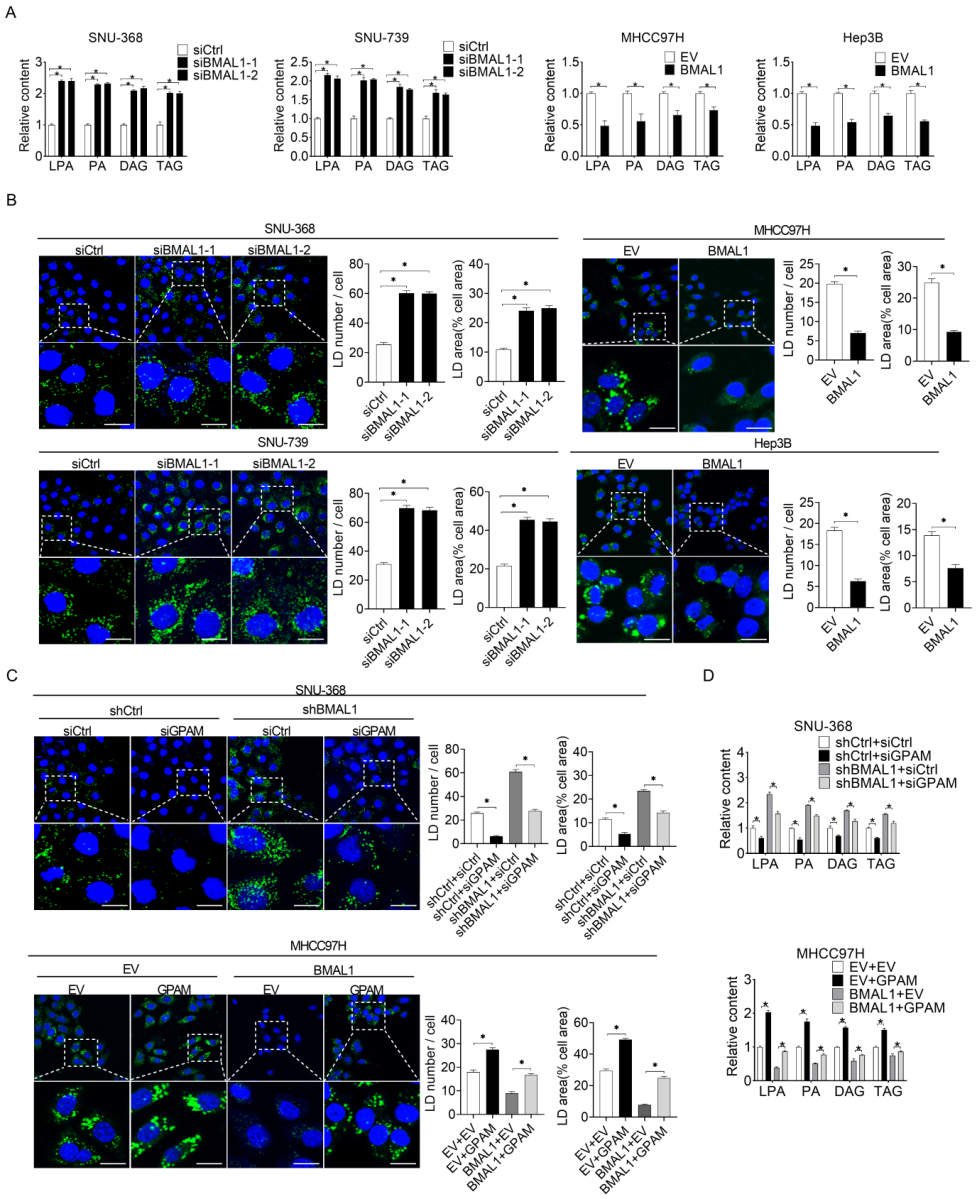
** BMAL1 suppressed glycerolipid synthesis in HCC by inhibiting GPAM levels. (A)** LPA, PA, DAG and TAG levels in treated SNU-368, SNU-739, MHCC97H and Hep-3B cells were evaluated by ELISA. **(B)** BODIPY 493/503 fluorescence dye staining for neutral lipids content in treated HCC cells. Scale bar, 25 µm. Average LD numbers per cell and proportion of cellular area occupied by LDs are quantified. **(C)** BODIPY 493/503 fluorescence dye staining for neutral lipid levels in treated HCC cells. Scale bars, 25 µm. Average LD numbers per cell and proportion of cellular area occupied by LDs are quantified. **(D)** LPA, PA, DAG and TAG in treated HCC cells were detected by ELISA. *p <0.05.

To elucidate the relationship between BMAL1 and GPAM-regulated glycerolipid synthesis, we quantified the intermediates in this metabolic pathway using mass spectrometry-based metabolomics analysis. Silencing of BMAL1 significantly increased the levels of triacylglycerol (TAG) lysophosphatidic acid (LPA), phosphatidic acid (PA), and diacylglycerol (DAG) in SNU-368 and SNU-739 cells. However, the levels of TAG, LPA, PA, and DAG decreased in MHCC97H and Hep3B cells overexpressing BMAL1 (**Figure 5A**). BMAL1 expression was also negatively associated with levels of LPA, PA, DAG and TAG in tumor tissues from 30 HCC patients** (Figure S5)**. Cellular staining with the lipophilic dye BODIPY 493/503 further showed that BMAL1 silencing elevated the intracellular levels of neutral lipids in SNU-368 and SNU-739, whereas BMAL1 overexpression diminished the intracellular levels of neutral lipids in HCC cells (**Figure 5B**). We then explored whether BMAL1 decreased the content of intracellular lipid through down-regulation of GPAM. Our results showed that GPAM silencing attenuated levels of neutral lipids and intracellular levels of LPA, PA, DAG and TAG increased by BMAL1 silencing in SNU-368 cells. In contrast, GPAM over-expression restored the content of neutral lipids and intracellular levels of LPA, PA, DAG and TAG decreased by BMAL1 over-expression in MHCC97H cells (**Figure 5C-5D**). These results indicate that BMAL1 decreases TAG and LPA levels in HCC by suppressing GPAM.

### BMAL1 suppresses HCC growth and metastasis by suppressing GPAM

**Figure 6:**
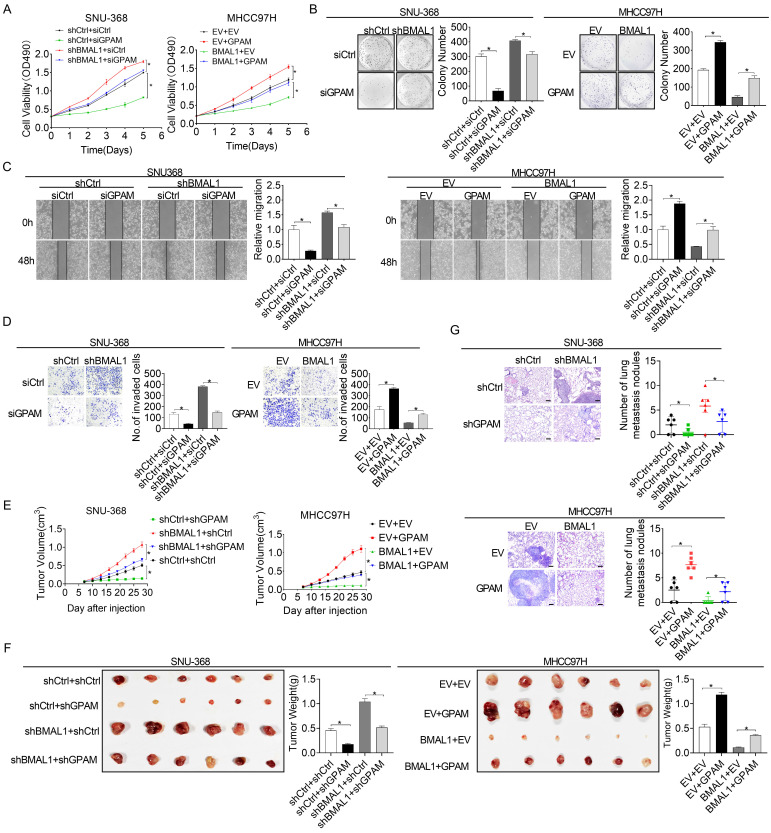
** BMAL1 suppressed HCC cell growth and metastasis by inhibiting GPAM. (A and B)** MTS and colony formation assays for treated SNU-368 and MHCC97H cells. **(C and D)** Scratch-wound-healing and matrigel invasion assays for migration and invasive potentials of specifically treated SNU-368 and SNU-739 cells. **(E)** Subcutaneous tumor-growth curves for HCC cells stably transfected with the expression vector encoding BMAL1 (n=6 mice/group). **(F)** Dissected subcutaneous tumors f (left panel), with their weights (right panel) (n=6 mice/group). **(G)** Number of lung metastasis nodules in each group (n=6 mice/group). Scale bar, 100 µm. *p <0.05.

Previous studies have reported that elevated intracellular lipids, such as TAG and LPA, play important roles in tumor growth and metastasis 20, 21. Therefore, we postulated that BMAL1 might suppress HCC growth and metastasis by transcriptionally inhibiting GPAM expression. As shown in the **Figure 6A and 6B**, silencing of GPAM attenuated HCC cell growth enhanced by BMAL1 silencing, whereas over-expression of GPAM restored HCC cell growth suppressed by BMAL1 over-expression. Cell migration and invasion assays also indicated that silencing of GPAM attenuated the metastasis ability of HCC cells promoted by BMAL1 knockdown, whereas over-expression of GPAM restored the metastasis ability suppressed by BMAL1 over-expression (**Figure 6C and 6D**). Similar results were also obtained from *in vivo* tumor growth and metastasis assays (**Figure 6E-6G**). These findings imply that BMAL1 represses HCC growth and metastasis by suppressing GPAM.

### Increased miRNA-494-3p contributes to the down-regulation of BMAL1 in HCC cells

**Figure 7:**
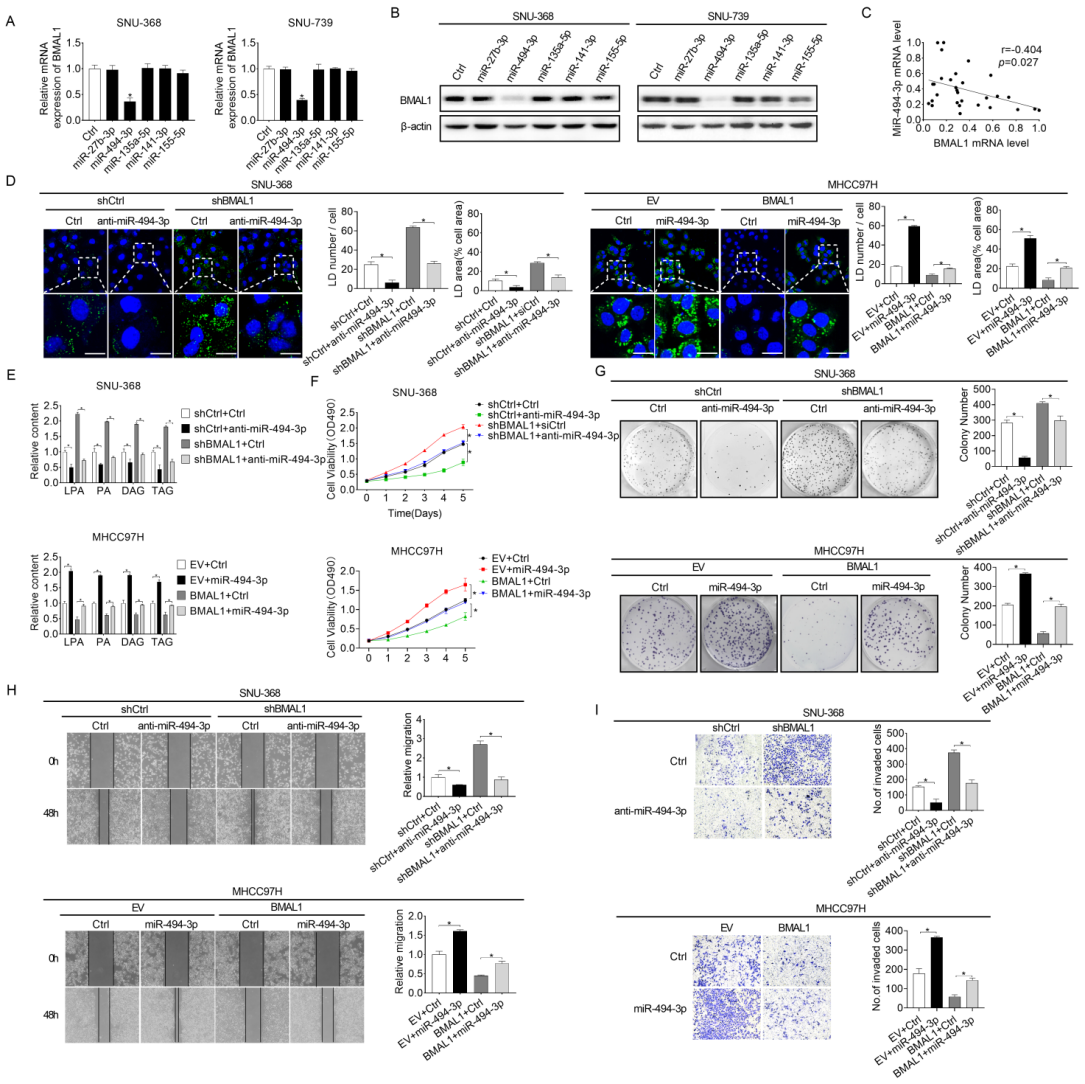
** Elevated miRNA-494-3p levels down-regulated BMAL1 in HCC cells. (A and B)** qRT-PCR and Western blot analyses of BMAL1 expressions in specifically treated HCC cells. **(C)** Relationship between mRNA expressions of miR-494-3p and BMAL1 in tumor tissues from 30 paired HCC individuals. **(D)** Neutral lipid levels in specifically treated SNU-368 and MHCC97H cells were assayed by BODIPY 493/503 dye staining. Scale bars, 25 µm. Average LD numbers per cell and proportion of cellular area occupied by LDs were quantified. **(E)** LPA, PA, DAG and TAG levels in specifically treated SNU-368 and MHCC97H cells were determined by ELISA. **(F and G)** MTS and colony formation assays for specifically treated SNU-368 and MHCC97H cells. **(H and I)** Scratch-wound-healing and matrigel invasion assays for specifically treated SNU-368 and MHCC97H cells. *p<0.05.

MicroRNAs play vital roles in gene expression regulation networks 22. Considering that BMAL1 is down-regulated at both protein and mRNA levels in HCC and the positive correlation between its mRNA expression and protein levels in individual patients, we analyzed microRNAs potentially involved in down-regulation of BMAL1 in HCC using an online microRNA Data Integration Portal (mirDIP)-based target prediction 23. Of the top five predicted miRNAs targeting BMAL1, only miR-494-3p transfection remarkably down-regulated BMAL1 expression at both protein and mRNA levels in HCC cells (**Figure 7A and 7B**). Besides, a negative relationship between mRNA expression levels of miR-494-3p and BMAL1 was reported in tumor tissues in 30 HCC individuals (**Figure 7C**). Furthermore, the effect of BMAL1 on glycerolipid metabolism (**Figure 7D and 7E**), and on growth and metastasis (**Figure 7F-7I**) was reversed by transfection with miR-494-3p mimics and anti-miR-494-3p in SNU-368 and MHCC97H cells, respectively.

To determine whether miR-494-3p down-regulates BMAL1 expression by directly binding to the 3'UTR of BMAL1 in HCC cells, we subcloned wild-type or mutant miR-494-3p binding sequences from the BMAL1 3'UTR into luciferase reporter vectors (**Figure S6A**). Then, one of these vectors combined with anti-miR-494-3p or miR-494-3p mimics were co-transfected into SNU-368 and MHCC97H cells, respectively. The luciferase activity of the BMAL1 wt 3'UTR vector was increased after the co-transfection with anti-miR-494-3p in MHCC97H cells (**Figure S6C**). SNU-368 cells co-transfected with the BMAL1 wild-type 3'UTR (wt 3'UTR) and miR-494-3p mimics showed significantly decreased luciferase activity (**Figure S6B**). However, no such changes were observed in SNU-368 and MHCC97H cells co-transfected with BMAL1 mutant 3'UTR (mt 3'UTR) vector and either anti-miR-494-3p or miR-494-3p mimics (**Figure S6B and S6C**). These results indicate that miR-494-3p directly regulates BMAL1 expression in HCC cells.

### REV-ERBα antagonist SR8278 has a therapeutic effect on HCC *in vitro* and *in vivo*

**Figure 8:**
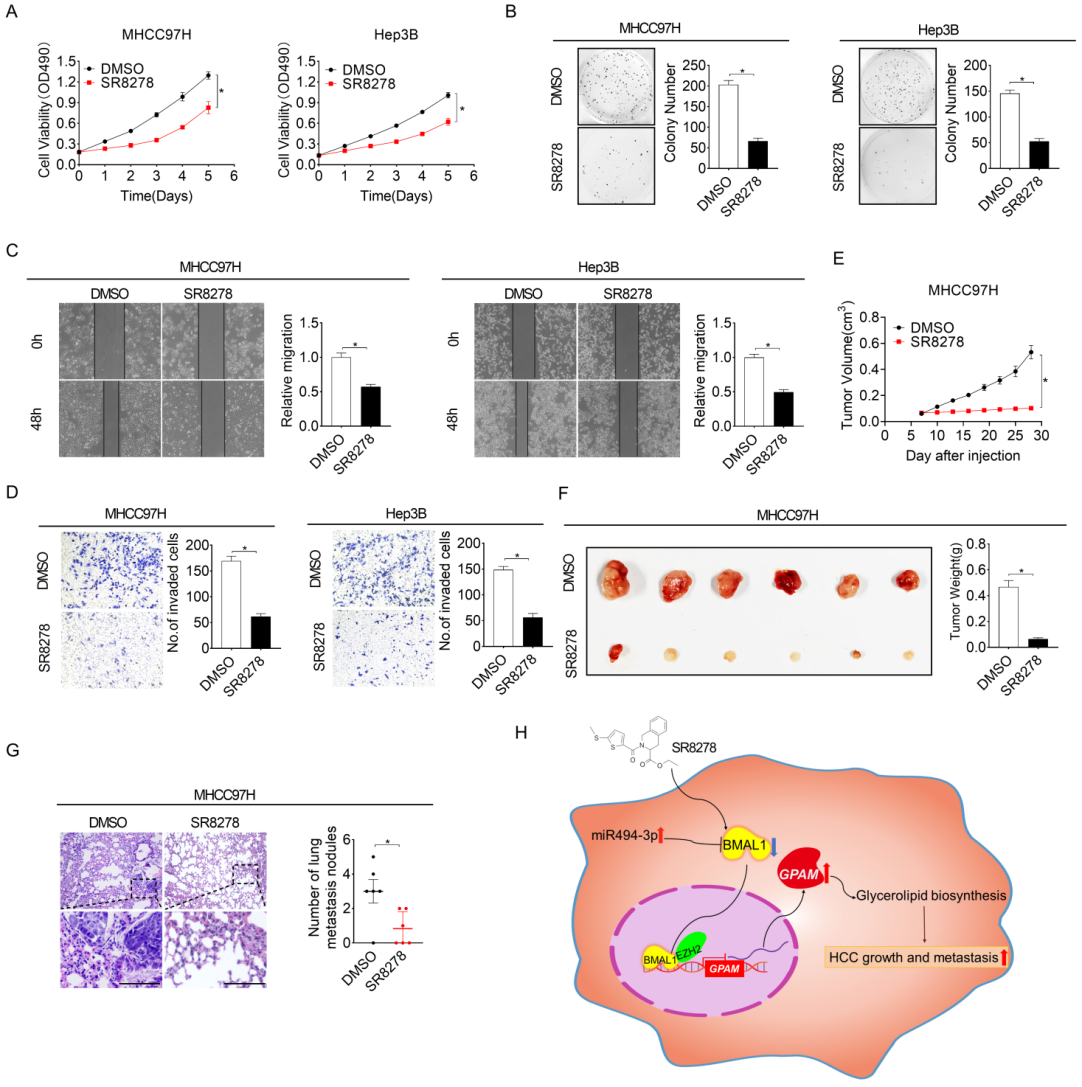
** The REV-ERBα antagonist SR8278 exhibited therapeutic effects against HCC *in vitro* and *in vivo*. (A and B)** MTS and colony formation assays were conducted for MHCC97H and Hep3B cells treated with 20 µM SR8278 for 24 h. **(C and D)** Scratch-wound-healing and matrigel invasion assays for SR8278-treated MHCC97H and Hep3B cells. **(E)** Subcutaneous tumor-growth curves for SR8278-treated MHCC97H cells (n=6 mice/group). **(F)** Dissected subcutaneous tumors (left panel) and their weights (right panel) (n=6 mice/group). **(G)** The number of lung metastasis nodules in each group (n=6 mice/group). Scale bar, 100 µm. *p <0.05.

SR8278 is a competitive synthetic antagonist of the nuclear receptor of REV-ERB, which negatively regulates BMAL1 transcription by directly docking to its promoter 24. To study the potential therapeutic influence of SR8278 in HCC, we first assessed the effect of SR8278 on the expressions of BMAL1 and GPAM. As shown in **Figure S7A and S7B**, SR8278 treatment markedly increased BMAL1 expression but decreased GPAM expression at both mRNA and protein levels in MHCC97H and Hep3B cells. Treatment with SR8278 decreased the content of neutral lipids and intracellular levels of LPA, PA, DAG and TAG in MHCC97H and Hep3B cells (**Figure S7C and S7D**). Notably, similar to the influence of BMAL1 overexpression on HCC growth and metastasis, SR8278 treatment reduced cell viability, colony formation, migration and invasion of MHCC97H and Hep3B cells (**Figure 8A-8D**). Additionally, *in vivo* influence of SR8278 treatment on growth and metastasis of HCC was also investigated. Our results indicated that SR8278 injection repressed the growth of xenograft tumors in MHCC97H cells (**Figure 8E-8F**) and lung metastasis of HCC cells (**Figure 8G**). Similar results were also obtained when HCC cells were treated with ROR agonist, SR1078 (**Figure S7E-S7L**). These data imply that forced expression of BMAL1 by SR8278 or SR1078 may be a prospective novel therapeutic approach for HCC.

BMAL1/ClOCK is a major component of the circadian molecular oscillator regulating E-box containing promoters 25. We thus explored whether CLOCK is involved in BMAL1-regulated GPAM expression in HCC. Knockdown of CLOCK obviously decreased the expression of GPAM both at mRNA and protein levels, whereas its overexpression increased GPAM expression (**Figure S7M and S7N**), suggesting that BMAL1 controls GPAM expression and subsequent lipid biogenesis in HCC cells by cooperating with CLOCK.

## Discussion

Emerging research evidence shows that disrupted expression level of circadian genes is strongly linked to tumorigenesis 26, 27. BMAL1 is a core circadian rhythm component. Its dysregulation has been shown to be closely associated aberrant metabolism in human diseases, including liver insulin resistance, hyperlipidaemia and atherosclerosis 28-30. Nevertheless, the biological functions of BMAL1, especially its role in lipid metabolism reprogramming in cancer cells, remain elusive. Here, we established that BMAL1 was significantly down-regulated in HCC cells, which is consistent with the results from several other cancer types, including pancreatic cancer, ovarian cancer, colorectal cancer and tongue squamous cell carcinoma 9, 10, 12, 31. In addition, the heterogenous down-regulation of BMAL1 across individual HCC cell lines and tumor tissues could be partially attributed to the variability in the expression of P2 isoform of hepatocyte nuclear factor 4 alpha (HNF4α), which has been shown to be over-expressed in HCC cells and represses the expression of BMAL1 32, 33. Nevertheless, in multiple types of human cancers such as pleural mesothelioma, high expression of BMAL1 has also been observed 34. BMAL1 was also reported to be highly expressed in diethylnitrosamine-induced hepatocellular carcinoma mice model 35. This contradiction may be explained by different roles of circadian genes in different tumor types and models, which further shows the complexity of the mechanisms of cancer development. Meanwhile, we found that BMAL1 down-regulation was linked to poor survival of HCC patients, indicating that BMAL1 may act as a potential new prognostic biosignature for patients with HCC.

We found that BMAL1 was commonly down-regulated in HCC. This implies a potential tumor-suppressive role for BMAL1 in HCC. With this connection, we investigated the function of BMAL1 in HCC both *in vitro* and *in vivo*. Our findings demonstrated that BMAL1 remarkably repressed HCC cells growth by suppressing G1-S cell cycle transition-mediated cell proliferation and inducting apoptosis. Moreover, subcutaneous xenograft model confirmed that BMAL1 remarkably attenuated tumorigenicity of HCC cells in nude mice. The significance of BMAL1 on HCC cell migration and invasion ability was also explored. Our results showed that BMAL1 repressed the migration and invasion of HCC cells by modulating key elements of EMT. The *in vivo* metastatic assay also illustrated that BMAL1 repressed lung metastasis in nude mice models. Consistent with our results in HCC, BMAL1 has also been documented as a tumor repressor in numerous other kinds of cancers. Thales et al. 26 showed that BMAL1 silencing results in enhanced proliferation of lung tumors. In colorectal cancer and tongue squamous cell carcinoma, increased expression of BMAL1 improved drug sensitivity of chemotherapeutics and treatment efficacy 9, 12. Overall, our results as well as those of others collectively support the conclusion that BMAL1 functions as a novel tumor suppressor in human cancers.

Lipid metabolism reprogramming is increasingly recognized as one of the hallmarks of cancer, supporting tumor growth and metastasis 36-38. Among all cancers, HCC is remarkably marked with enhanced biosynthesis of triacylglycerols, phospholipids and cholesterol, which not only supply cancer cells with the substrates required for biomass generation, but also provide signaling molecules and energy sources for cancer cells to proliferate and metastasize to distant organs 39, 40. Nevertheless, mechanisms through which cancer cells rewire their fatty acid metabolism remain largely uncharacterized. Mounting evidence has demonstrated that dysregulation of circadian genes contributes to various metabolic syndromes, such as obesity and insulin resistance 41, 42. Nonetheless, the functions of the core circadian genes in metabolism reprogramming in cancer cells are still unclear. Recently, we have demonstrated that NPAS2, a commonly up-regulated circadian gene in HCC, participates in the progression of HCC by regulating glucose metabolism reprogramming 43. Nevertheless, whether dysfunction of circadian genes also contributes to abnormal tumor lipid metabolism remains unclear. Here, we showed that lipid metabolism in HCC cells was modulated by the core circadian rhythm component BMAL1, which markedly contributed to the development of HCC. Similar to our data in HCC, a previous study has reported that circadian misalignment induces liver metabolic disorders such as nonalcoholic fatty liver disease (NAFLD), which is typified by excessive fat accumulation 44. Besides, in human night-shift workers, systemic, as well as liver metabolic disorders have been frequently reported 45. These results collectively indicate that circadian disruption plays crucial roles in the development and progression of metabolic diseases, including human cancers.

Glycerol-3-phosphate acyltransferase mitochondrial (GPAM) is a pivotal enzyme in the lipid biosynthesis of triacylglycerols and phospholipids 46. Upregulation of GPAM and its oncogenic functions have been proved in several cancer types 21, 47. For example, the increased expression of GPAM has been revealed in breast cancer 48. In ovarian cancer cells, GPAM overexpression suppresses both tumor cell growth and migration 21. Consistently, we also demonstrated that BMAL1 down-regulation promoted HCC growth and metastasis by transcriptionally up-regulating GPAM. As a well-known transcription factor, BMAL1 has been shown to exert its function by transcriptionally activating its downstream target genes. Nevertheless, the present study showed that BMAL1 transcriptionally down-regulated GPAM expression by cooperating with EZH2. Actually, a previous study also has demonstrated that BMAL1 transcriptionally down-regulates its downstream target TERT in an EZH2-dependent manner in tongue squamous cell carcinoma cells 12. Additionally, the protein-protein interaction between BMAL1 and EZH2 has also been revealed using co-IP assay 19. These findings indicate that EZH2 is a crucial negative cofactor of BMAL1 in transcriptional regulation of its downstream target genes. Besides, we further demonstrated that BMAL1 transcriptionally activated GPAM by directly binding to the E-box in GPAM promoter region at sites nt-179 to -174. As a crucial product of GPAM, lipid lysophosphatidic acid (LPA) has been associated with cell proliferation, metabolic regulation and sustainability of cellular homeostasis 49. At high levels, LPA participates in the onset as well as progression of several kinds of human cancers 50. For example, Leblanc et al. 51 reported that platelet derived LPA contributed to the bone metastasis of breast cancer. Magkrioti et al. 52 also demonstrated that LPA participated in lung carcinogenesis. Increased LPA level has also been shown to be closely linked to poor prognosis in renal cancer patients 20. We additionally demonstrated that the anti-tumor effect of BMAL1 was dependent on the down-regulation of GPAM. These results collectively indicate that GPAM dysfunction-mediated lipid metabolism reprogramming plays contribute to cancer development.

MiRNA-494-3p is an onco-miRNA that is frequently over-expressed in various tumor types, including HCC. Zhang et al. 53 documented that miR-494-3p is up-regulated in colorectal cancer and its up-regulation promotes tumorigenesis and cell proliferation. Ma et al. 54 revealed that miR-494-3p is up-regulated in follicular lymphomas. In HCC, Chuang et al. 55 and Lin et al. 56 demonstrated the remarkable up-regulation of miR-494-3p, which contributes to tumor cell metastasis by silencing multiple genes involved in cell invasion. These findings imply that miR-494-3p is a crucial onco-miRNA in different cancer types. We established that miR-494-3p is involved in down-regulation of BMAL1 in HCC, in tandem with another finding in HEK293 cells that showed that transfection with miR-494-3p markedly suppressed luciferase-reported BMAL1 3' UTR activities 57. However, studies should determine whether other genetic and epigenetic factors also lead to down-regulation of BMAL1 in HCC.

BMAL1 is a transcription factor that is challenging to target 8. The circadian machinery has an independent negative feedback loop 58, for which pharmacologic agents have been developed as antagonists or agonists. Among them, SR8278 is a competitive synthetic antagonist of nuclear receptor of REV-ERB, which negatively regulates BMAL1 transcription by directly binding its promoter 24, 59, 60. Due to its safety and protective benefits, SR8278 is a potential therapeutic drug for Duchene muscular dystrophy 61. In this study, we established that SR8278 markedly suppressed HCC cell growth and metastasis *in vitro* and in *in vivo* xenograft models. Similarly, activation of BMAL1 using a ROR agonist SR1078 markedly reduced MHCC97H and Hep3B cell viabilities, colony formation, migration and invasion. Therefore, SR8278 and SR1078 are potential therapeutics for HCC. Although they are easy to develop and measure, cell line-based xenografts are limited in that tumor genetics and histology are not representative of human tumors. Thus, other tumor mice models that are more useful for *in vivo* pharmacological testing, such as patient-derived xenograft (PDX) and stelic animal model (STAM) models, should also be used to evaluate the therapeutic significance of SR8278 and SR1078 for HCC treatment.

In conclusion, BMAL1 is down-regulated in HCC. BMAL1 inhibits tumor progression by suppressing glycerolipid metabolism through transcriptional inhibition of GPAM expressions in an EZH2 dependent way. Moreover, activation of BMAL1 by treatment with SR8278 markedly suppressed HCC progression, suggesting a prospective treatment approach for HCC.

## Supplementary Material

Supplementary materials and methods, figures and tables.

## Abbreviations

BMAL1: brain and muscle arnt-like protein 1
EZH2: enhancer of zeste 2 polycomb repressive complex 2 subunit
GPAM: glycerol-3-phosphate acyltransferase mitochondrial
HCC: hepatocellular carcinoma
IHC: immunohistochemistry
qRT-PCR: quantitative real-time reverse transcription PCR
