# Supplementary material for: Down-regulation of BMAL1 by MiR-494-3p Promotes Hepatocellular Carcinoma Growth and Metastasis by Increasing GPAM-mediated Lipid Biosynthesis
Source: Int J Biol Sci. 2022 Oct 18;18(16):6129–44. doi: 10.7150/ijbs.74951 (PMC9682529; doi:10.7150/ijbs.74951)
Supplement: Supplementary file 1 — Supplementary materials and methods, figures and tables. [file ijbsv18p6129s1.pdf]

**Supplemental information**

**Supplemental materials and methods**

**HCC cell lines and tissues**

The human HLE, HLF, Hep3B, SNU-368, SNU-398, SNU-739, Huh7, MHCC97H and THLE-2 HCC cell lines were routinely cultured in Dulbecco's DMEM or RPMI-1640 medium enriched with 10% fetal bovine serum (HyClone Laboratories), as well as 100 μg/mL of streptomycin under humid 5% $CO_2$ conditions at 37°C. Moreover, 247 paired (30 paired for qRT-PCR and Western blotting assays; 217 paired for IHC staining assay) human HCC tumor and peritumor tissue samples as well as 36-unpaired primary and metastatic HCC tissue samples were acquired from the Xijing Hospital of the Fourth Military Medical University in Xi'an, China, as previously described[1]. The Ethical Committee of the Fourth Military Medical University (Xi'an, China) approved this study and written consents were obtained from all participants.

**qRT-PCR**

Total RNA from HCC cell lines or tissues were extracted using the Trizol Reagent (Invitrogen, 15596018) and reverse transcribed into cDNA using a PrimeScript RT Reagent kit, as per the manufacturer's protocol. qRT-PCR was peformed using a SYBR Green PCR Kit (Takara, 639676). The $2^{-\triangle\triangle CT}$ approach was used to determine relative expressions of target genes. The housekeeping-gene (β-actin) was the internal standard. The primers used in this assay are shown in **Supplementary Table 2**.

**Western blotting**

Total proteins were purified from HCC cell lines or tissues as described previously[2]. Protein concentrations were determined via the BCA assay (Bio-Rad Laboratories). Equivalent protein amounts were resolved on SDS–polyacrylamide gels, electroblotted onto PVDF membranes and incubated overnight at 4 °C in the presence of specific primary antibodies. Relative protein expressions of target genes were determined after incubation at room temperature for 2 h in the presence of appropriate secondary horseradish-peroxidase-labeled antibodies. Visualization was performed via an enhanced chemiluminescence assay. The primary antibodies in this assay and their working concentrations are shown in **Supplementary Table 2**.

**H&E and immunohistochemistry staining**

H&E and immunohistochemistry staining were performed as previously described[3]. Immunostains were scored based on positive staining cell proportions and staining intensity[3]. Primary antibodies in this assay and their working concentrations are specified in **Supplementary Table 2**.

**Flow cytometry analysis for cell cycle and apoptosis**

For cell cycle analysis, HCC cells with different treatments were fixed in 70% ethanol at 4°C overnight, stained with propidium iodide (BestBio, shanghai, China) at room temperature for 30 min, and assessed by flow cytometry (Beckman, Fullerton, CA). For cell apoptosis analysis, the FITC-Annexin V and PI Apoptosis Kit (F6012, US EverbrightInc) was used. A total of 5 μl ANXA5-FITC and 5 μl PI were introduced into HCC cells and incubated at room temperature for 20 min in the dark. Cells were rinsed thrice using PBS and analyzed by flow cytometry (Beckman,

Fullerton, CA).

**Wound-healing, cell migration and matrigel invasion assays**

To determine cell migration abilities, a pipette tip was used to scratch the middle of wells when cells in the 6-well plates had achieved a 85% confluence. Imaging of wound-closures was performed using a light Olympus microscope at 0 and 48 h after scratching. Relative migrations of differentially treated HCC cells were determined using the Image J software. For matrigel invasion assessment, transwell chambers coated with the matrigel matrix (BD Science) were used. Briefly, $1 \times 10^5$ cells were loaded into the upper chamber of each well. After 48 h of incubation in a 5% $CO_2$ atmosphere at 37°C, cells that had invaded the lower chamber were fixed in 4% formaldehyde for 10 min and stained with 0.1% crystal violet for 10 min at room temperature. The number of invaded cells in each group was determined by light microscopy.

**Reporter plasmids and site-directed mutagenesis**

Promoter sequences of GPAM were abstracted from the UCSC Genome Browser. Truncated portions of the GPAM promoter amplified by PCR at selected regions were inserted into pGL3-Basic vectors (Promega, Madison, WI). The primer sequences used in this study are indicated in supplementary Table 1. Site-directed mutagenesis was performed using a Q5 Site-Directed Mutagenesis Kit (NEB, E0552S) as instructed by the manufacturer.

**Immunofluorescence**

For double immunofluorescence staining of BMAL1 and EZH2, HCC cells were

plated in the culture dish and incubated with primary BMAL1 and EZH2 antibodies at 4°C overnight. After rinsing twice in PBS, they were respectively incubated with 488-labelled goat anti-rabbit or 594-labelled goat anti-mouse secondary antibodies. Counterstaining of the nucleus was performed using 4', 6-diamidino-2-phenylindole (DAPI) followed by imaging using an Olympus FV 1000 laser-scanning confocal microscope.

**ELISA for determination of lipid levels**

The amounts of glycerolipid synthesis products (LPA, PA, DAG, TAG) in HCC cells were measured using the avidin biotin system (ABS) antibody sandwich ELISA method. The LPA, PA, DAG, and TAG kits (human, 96-well) were purchased from mlbio (shanghai). Based on the instructions, intracellular components of HCC cells ($1\times10^6$cells/ml) were extracted and quantitated by ELISA. Assays were performed as described [4, 5].

***In vivo* tumorigenicity and metastatic assays**

To assess the *in vivo* tumor growth, $1x10^7$ HCC cells with varied treatments were subcutaneously administered into the flanks of four to five-week-old male BALB/c nude mice (n=6 per group). Then, tumor volumes were determined using a Vernier caliper every week. At 5 weeks after cell injections, mice were sacrificed, tumors were harvested and their weights determined. The ethical committee of the Fourth Military Medical University for animal research approved the use of animals in this study. When tumors were 3 to 4mm in diameter, SR8278 at 0.5 mg/mice was administered in each tumor twice a week.

For the *in vivo* metastatic assay, $5\times10^6$ HCC cells with varied treatments were intravenously administered into four to five-week-old male BALB/c nude mice via their tail veins (n=6 per group). Mice were sacrificed at two months after cell administration and their lungs collected for H&E staining. Metastatic tumor nodules in lungs were counted. SR8278 at dose of 0.5 mg/mice was administered into each mouse twice a week by intraperitoneal injection after operation.

**Silencing and forced expressions of target genes**

To transiently silence BMAL1, small interference RNAs (siRNAs) targeting BMAL1 were transfected into HCC cells using the lipofectamine 2000 reagent (Invitrogen) as per the manufacturer's instructions. A pSilencer™ 3.1-H1 puro vector (Ambion) was used to construct the shRNA targeting BMAL1. To construct the BMAL1 over-expression vector, the BMAL1 coding sequence was amplified and cloned into a pcDNA™3.1(C) vector (Invitrogen).

**Luciferase assay**

Luciferase assays were performed as previously described[1]. The HCC cells were co-transfected with 4 μg of GPAM promoter constructs and Renilla luciferase expressing control vector and grown for 48 h. Cells were lysed using the ice-cold lysis buffer after which luciferase enzyme activities were assessed by Dual Luciferase Reporter Assay (Promega, E1910), as instructed by the manufacturer. A Luminoscan Ascent MicroplateLuminometer (Thermo Scientific) was used to determine the relative light units. Luciferase activities were standardized to renilla luciferase enzyme activities.

**Chromatin immunoprecipitation (ChIP)-PCR assay**

The ChIP assay was performed using a ChIP assay kit (Cell Signaling, #9005), as per the manufacturers' protocol. The HCC cells were fixed in 1% formaldehyde and disrupted in the lysis buffer. Subsequently, samples were sonicated to degrade the nuclear membranes and the supernatants collected. Then, immune-precipitations of chromatin by an anti-BMAL1 antibody (1:100) or equivalent quantities of normal rabbit IgG were performed. DNA was isolated and PCR amplified using primer pairs within the modulatory site of GPAM, sequences of which are shown in **Supplementary Table 1**.

**Co-immunoprecipitation (Co-IP) assay**

For co-IP assays, HCC cells were lysed in ice-cold lysis buffer and incubated overnight at 4°C with 25 µl protein A beads (Santa Cruz) enriched with anti-BMAL1 (1:500) and anti-GPAM (1:300) antibodies. After rinsing 3 times using cold washing buffer, immunoprecipitated samples were eluted by heating in a loading buffer at 100 °C for 5 mins, followed by immunoblotting (WB) for analysis.

**Statistical analysis**

Data are shown as mean ± SEM. The SPSS software (17.0 version, Chicago, IL) was used for analyses, with $p < 0.05$ as the threshold for significance (*). Overall and recurrence-free survival curves were computed using the Kaplan-Meier approach. Two-tailed student's t-test and one-way ANOVA followed by Tukey's post-hoc test were used for comparisons of means between and among groups, respectively. Relationships between measured variables were determined by Spearman rank

correlation analysis.

**Supplemental figures**

**Figure S1:** Western blot analysis for BMAL1 expressions in 30 paired HCC and adjacent non-tumor tissues.

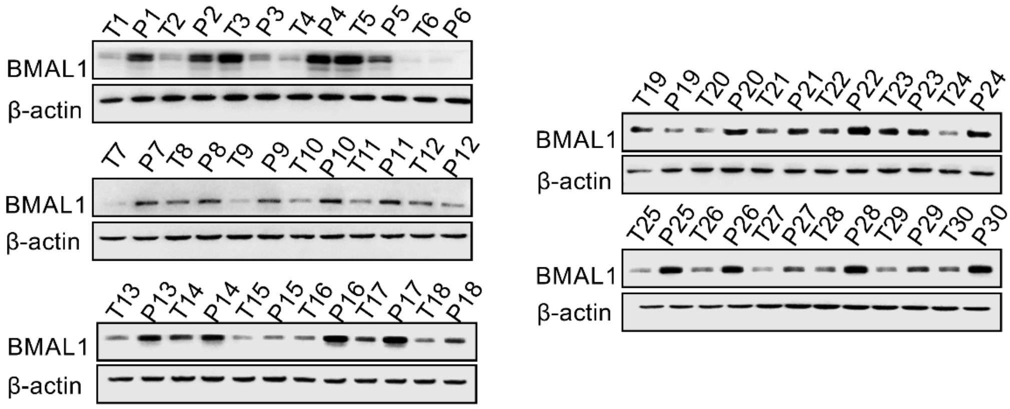

**Figure S2:** (A and B) Knockdown of BMAL1 in SNU-368 and SNU-739 cells was confirmed by qRT-PCR (A) and Western blot (B) analysis. (C) EdU incorporation assay for SNU-368 and SNU-739 cells with BMAL1 knocked-down (Scale bar, 50 μm). (D and E) Cell cycle distribution and apoptosis of treated SNU-368 and SNU-739 cells was evaluated by flow cytometry. (F and G) Western blot analysis for key regulators involved in regulation of cell cycle distributions and apoptosis of SNU-368 and SNU-739 cells with BMAL1 knocked-down. (H) Western blot analysis for expressions of EMT markers in treated SNU-368 and SNU-739 cells. (I) IHC staining of Ki-67 in tumor tissues from subcutaneous xenografts. Scale bar, 100 μm. (J) TUNEL assay for tumor tissues from subcutaneous xenografts. Scale bar, 100 μm.

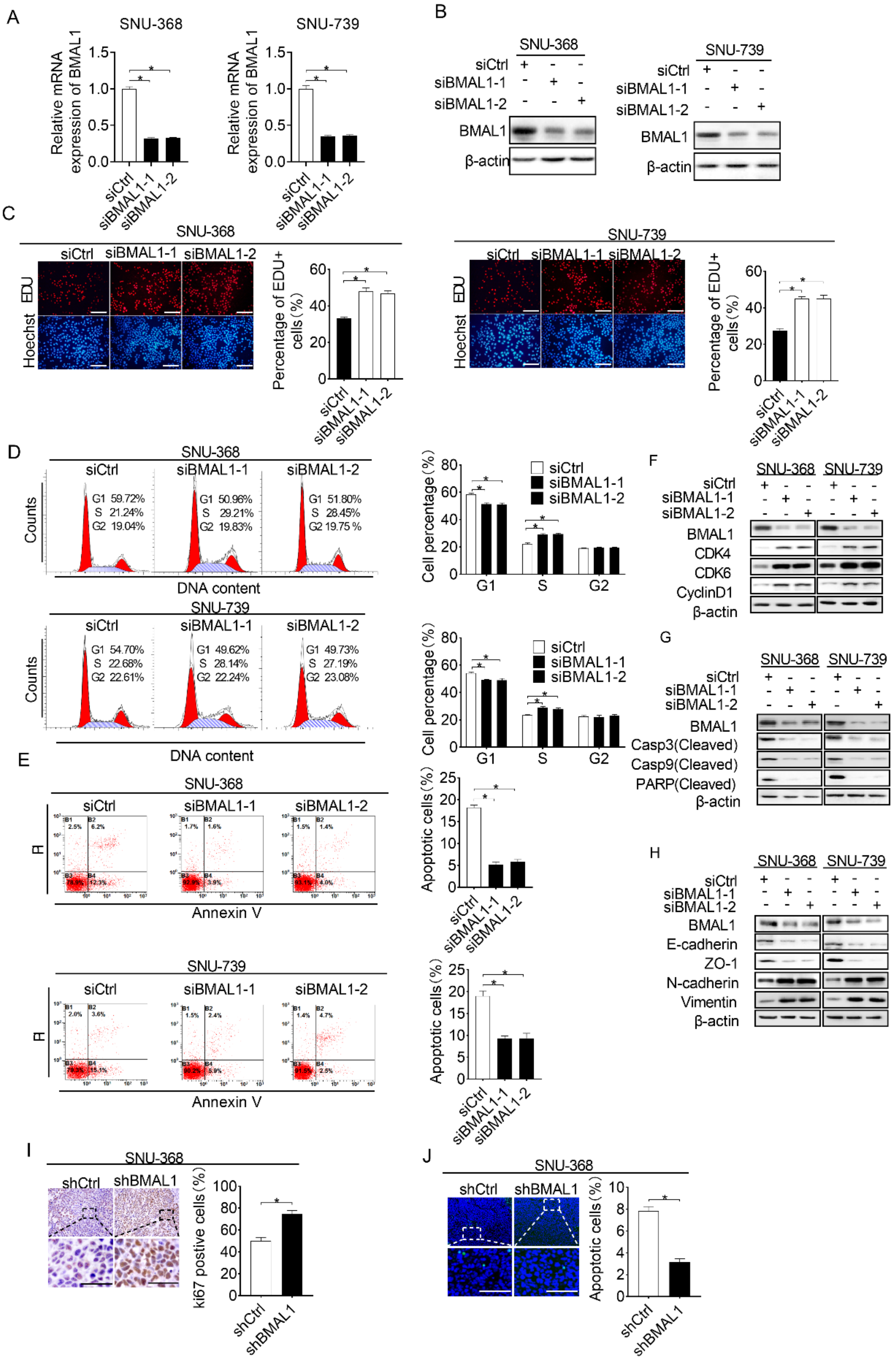
A
SNU-368
SNU-739
Relative mRNA expression of BMAL1
1.5
1.0
0.5
0.0
siCtrl
siBMAL1-1
siBMAL1-2
B
SNU-368
SNU-739
siCtrl
siBMAL1-1
siBMAL1-2
BMAL1
β-actin
C
SNU-368
SNU-739
siCtrl siBMAL1-1 siBMAL1-2
EDU
Hoechst
Percentage of EDU+ cells (%)
60
40
20
0
D
SNU-368
siCtrl
siBMAL1-1
siBMAL1-2
Counts
G1 59.72%
S 21.24%
G2 19.04%
G1 50.96%
S 29.21%
G2 19.83%
G1 51.80%
S 28.45%
G2 19.75 %
DNA content
SNU-739
G1 54.70%
S 22.68%
G2 22.61%
G1 49.62%
S 28.14%
G2 22.24%
G1 49.73%
S 27.19%
G2 23.08%
DNA content
Cell percentage (%)
80
60
40
20
0
G1
S
G2
E
SNU-368
siCtrl
siBMAL1-1
siBMAL1-2
PI
Annexin V
SNU-739
Apoptotic cells (%)
25
20
15
10
5
0
F
SNU-368
SNU-739
BMAL1
CDK4
CDK6
CyclinD1
β-actin
G
BMAL1
Casp3(Cleaved)
Casp9(Cleaved)
PARP(Cleaved)
β-actin
H
BMAL1
E-cadherin
ZO-1
N-cadherin
Vimentin
β-actin
I
SNU-368
shCtrl
shBMAL1
ki67 postive cells (%)
100
80
60
40
20
0
J
SNU-368
shCtrl
shBMAL1
Apoptotic cells (%)
10
8
6
4
2
0

**Figure S3:** (A and B) Overexpressions of BMAL1 in MHCC97H and Hep3B cells were confirmed by qRT-PCR (A) and Western blot (B) analyses. (C) EdU incorporation assay for MHCC97H and Hep3B cells with BMAL1 overexpressed (Scale bar, 50 μm). (D and E) Cell cycle distributions and apoptosis of MHCC97H and Hep3B cells with BMAL1 overexpressed were evaluated by flow cytometry. (F and G) Western blot analysis for key regulators of cell cycle distributions and apoptosis of MHCC97H and Hep3B cells with BMAL1 overexpressed. (H) Western blot analysis for expressions of EMT markers in MHCC97H and Hep3B cells with BMAL1 overexpressed. (I and J) Ki-67 IHC staining and TUNEL assays of tumor tissues from subcutaneous xenografts (Scale bar, 100 μm).

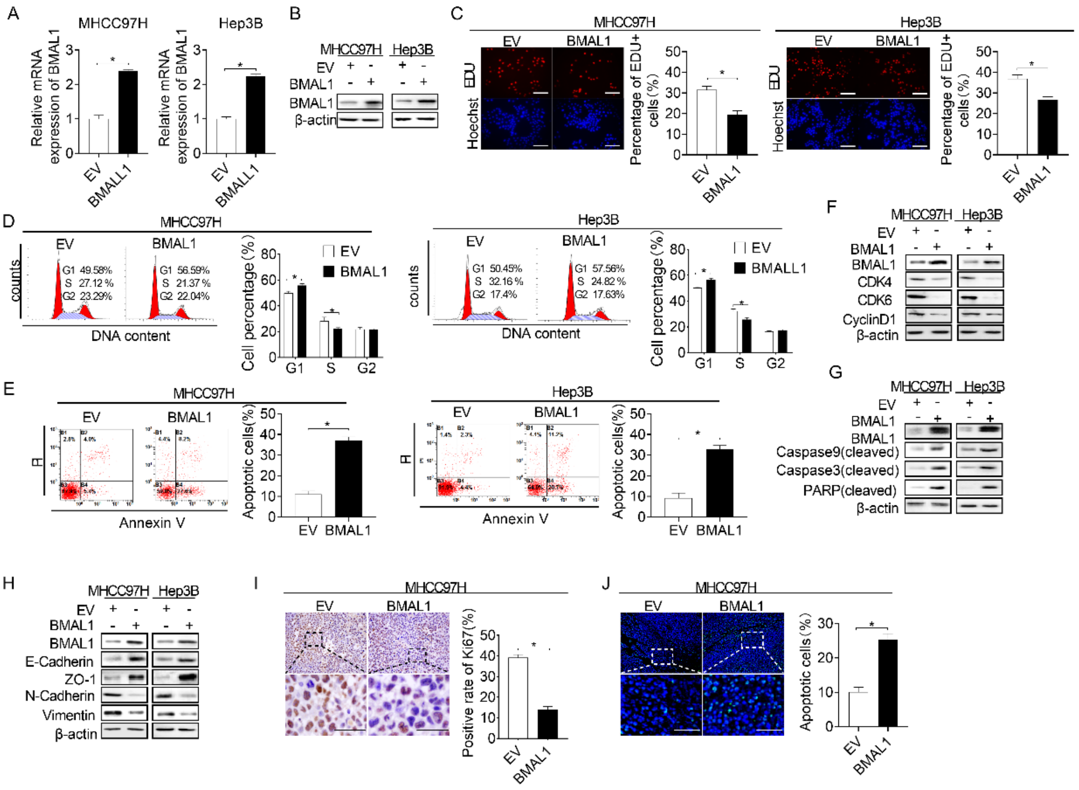

**Figure S4: (A)** IHC-staining for GPAM in 36-unpaired primary and metastatic HCC tissues. (B) Correlations between protein expression levels of BMAL1 and GPAM in 36-unpaired primary and metastatic HCC tissues. *p < 0.05. Scale bar: 100 μm. (C) Expressions of GPAM were assessed by qRT-PCR (left) and western blot (right) assays (n=217). (D) Correlations between protein expressions of BMAL1 and GPAM in HCC and normal hepatic cell lines. (E) Prognostic value of GPAM was determined by IHC staining (n=217).

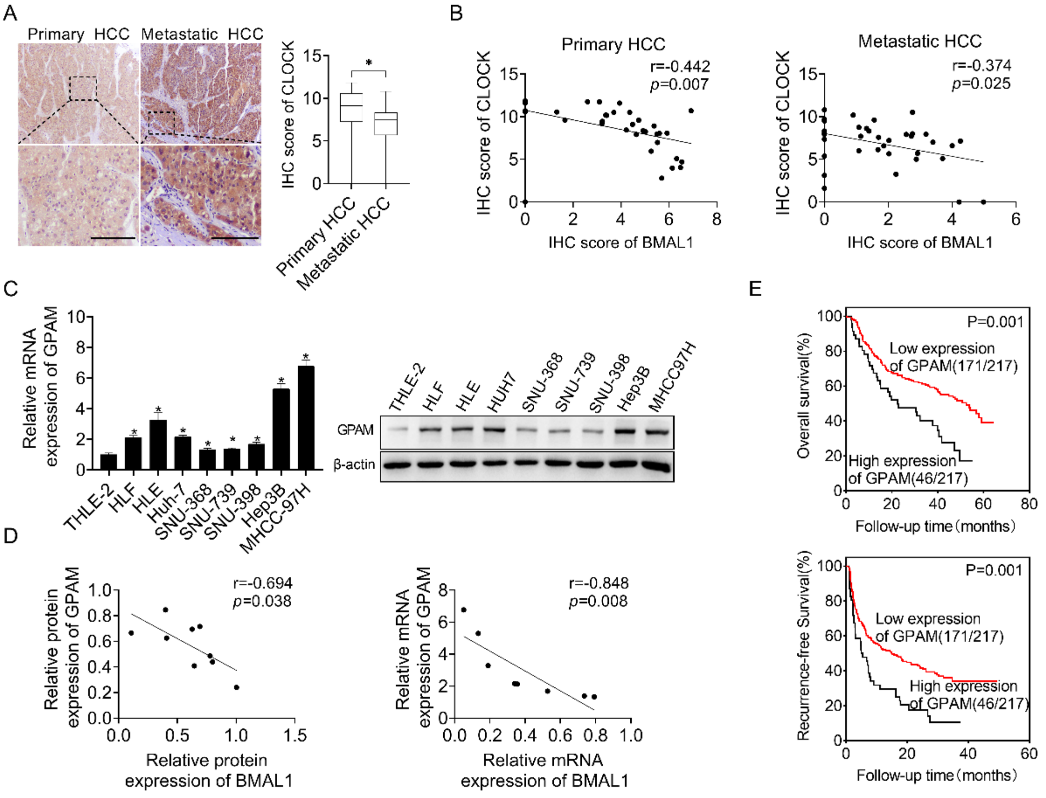

**Figure S5:** Correlations between BMAL1 expressions and levels of LPA, PA, DAG and TAG in tumor tissues from 30 HCC patients.

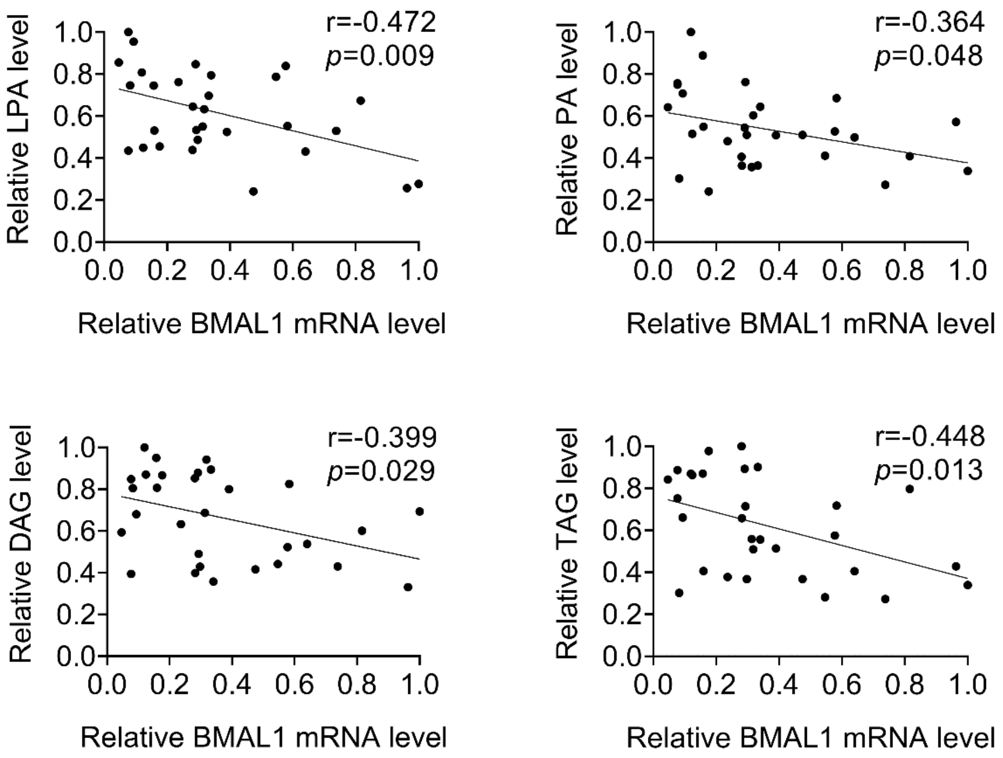

**Figure S6: BMAL1 is a target gene for miR-494-3p.** (A) Predicted binding sequence for miR-494-3p within the BMAL1 3'UTR. Seed sequences are highlighted. (B) Luciferase reporter assay for SNU-368 cells co-transfected with wild-type or mutant miR-494-3p and anti-miR-494-3p. (C) Luciferase reporter assay for MHCC97H cells co-transfected wild-type or mutant miR-494-3p and miR-494-3p mimics. NS: no significant difference.

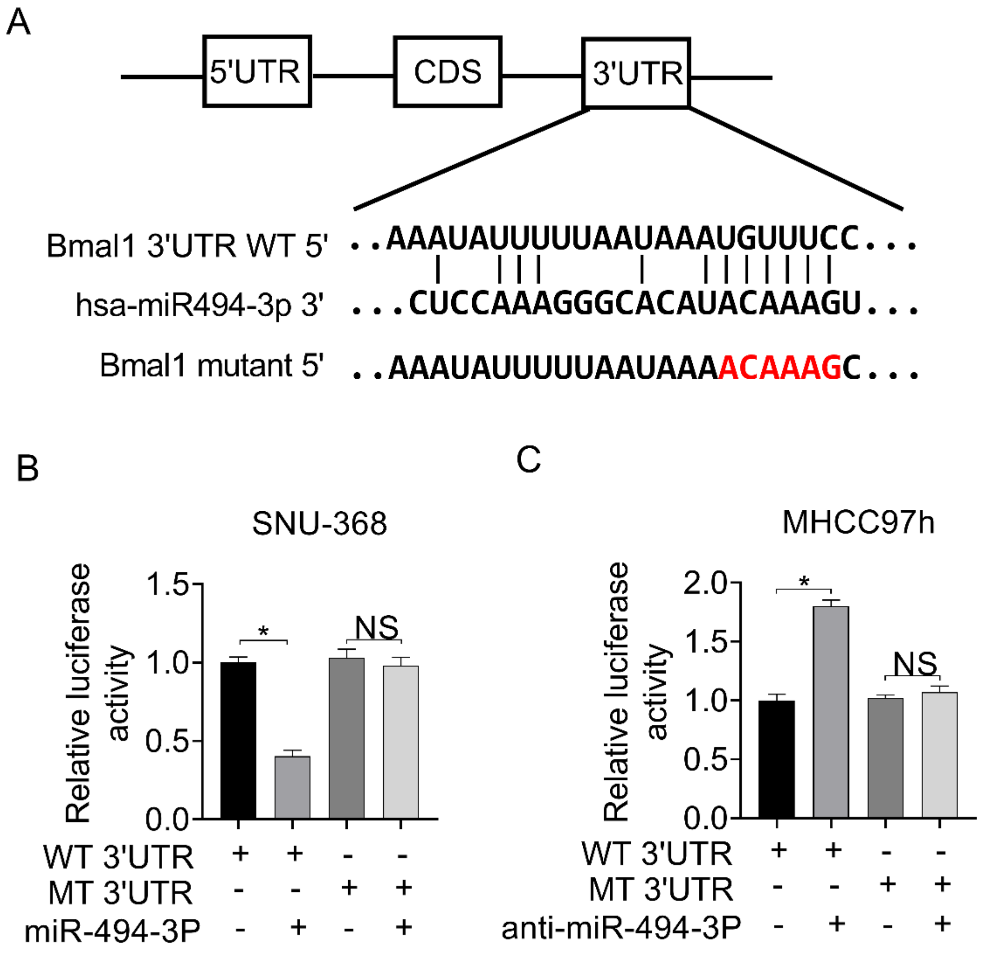

**Figure S7:** (A and B) qRT-PCR and western blot analysis for expressions of BMAL1 and GPAM in MHCC97H and Hep3B cells treated with the REV-ERBα antagonist, SR8278. (C) Levels of neutral lipids in MHCC97H and Hep3B cells treated with the REV-ERBα antagonist, SR8278, were detected by fluorescence BODIPY 493/503 dye staining. Scale bars, 50 μm. Average number of LDs per cell and percentage of cellular area occupied by LDs were quantified. (D) Levels of G3P, PA, DAG, TAG and LPA in MHCC97H and Hep3B cells treated with SR8278 were detected by ELISA. (E and F) qRT-PCR and western blot analysis for expressions of BMAL1 and GPAM in MHCC97H and Hep3B cells treated with a ROR agonist, SR1078, at a concentration of 10 μM for 24 h. (G) Levels of neutral lipids in MHCC97H and Hep3B cells treated with SR1078 were determined by fluorescence BODIPY 493/503 dye staining. Scale bars, 50 μm. Average number of LDs per cell and percentage of cellular area occupied by LDs were quantified. (H) Levels of G3P, PA, DAG, TAG and LPA in MHCC97H and Hep3B cells treated with SR1078 were detected by ELISA. (I and J) MTS and colony formation assays were conducted for MHCC97H and Hep3B cells exposed to SR1078. (K and L) Scratch-wound-healing and matrigel invasion assays for MHCC97H and Hep3B cells treated with SR1078.

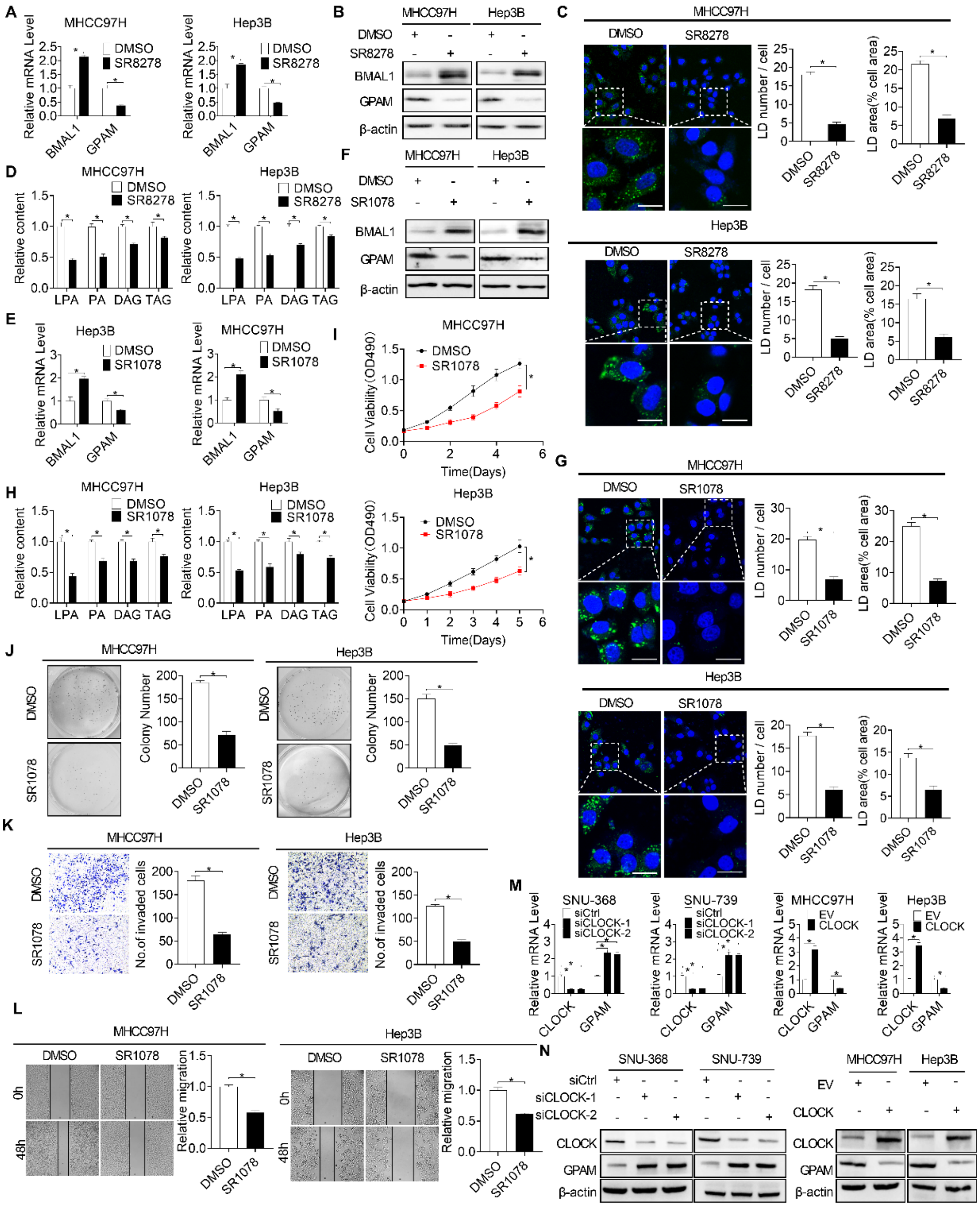
A
MHCC97H
Hep3B
Relative mRNA Level
DMSO
SR8278
BMAL1
GPAM
B
MHCC97H
Hep3B
DMSO
SR8278
BMAL1
GPAM
β-actin
C
MHCC97H
DMSO
SR8278
LD number / cell
LD area(% cell area)
Hep3B
D
MHCC97H
Hep3B
Relative content
LPA
PA
DAG
TAG
E
Hep3B
MHCC97H
SR1078
F
DMSO
SR1078
I
MHCC97H
Cell Viability(OD490)
Time(Days)
G
MHCC97H
Hep3B
H
J
Colony Number
K
No.of invaded cells
L
0h
48h
Relative migration
M
SNU-368
SNU-739
siCtrl
siCLOCK-1
siCLOCK-2
EV
CLOCK
N
SNU-368
SNU-739
MHCC97H
Hep3B
siCtrl
siCLOCK-1
siCLOCK-2
EV
CLOCK
GPAM
β-actin

**Supplementary Tables**

**Table S1: Sequences of primers, siRNAs and miRNAs used in this study.**

**1. Primers used in q-PCR analysis**

| | | |
|---|---|---|
| *BMAL1* | forward primer | GGATGTGACCGAGGGAAGAT |
| | reverse primer | CGTCGTGCTCCAGAACATAAT |
| *GPAM* | forward primer | GATGTAAGCACACAAGTGAGGA |
| | reverse primer | TCCGACTCATTAGGCTTTCTTTC |
| *MGLL* | forward primer | GCTCTCGAGGCCGCCATGCCAGAGGAAAGTTCC |
| | reverse primer | AGCTGAATTCTCAGGGTGGGGACGCAGTTCCTG |
| *DGAT2* | forward primer | ATTGCTGGCTCATCGCTGT |
| | reverse primer | GGGAAAGTAGTCTCGAAAGTAGC |
| *PPAP2B* | forward primer | TGAGAGCATCAAGTACCCACT |
| | reverse primer | ACGTAGGGGTTCTGAATCGTC |
| miR-494-3p | forward primer | ACACTCCAGCTGGG TGAAACATACACGGGA |
| | reverse primer | CTCAACTGGTGTCGTGGAGTCGGCAATTCAGTTG |
| U6 | forward primer | CTCGCTTCGGCAGCACA |
| | reverse primer | AACGCTTCACGAATTTGCGT |
| *GAPDH* | forward primer | GGAGCGAGATCCCTCCAAAAT |
| | reverse primer | GGCTGTTGTCATACTTCTCATGG |
| *CLOCK* | forward primer | GCTCGAGGGTGGCTGGGAGTTTTGATTG |
| | reverse primer | GCTCTAGACACGTTTAGAAGGCATGTGAGTTAC |

**2.Primers for *GPAM* promoter construct**

| | | |
|---|---|---|
| (-2076/+86) GPAM | forward primer: | CGGGATCCCGCCTCCCGTCAGGTTTTTTACTG |
| (-929/+86) GPAM | forward primer: | CGGGATCCCGCTCAAGGCAATCATAGGGC |
| (-428/+86) GPAM | forward primer: | CGGGATCCCGCGCACTCAGCCACTTTGGTA |
| (-102/+86) GPAM | forward primer: | CGGGATCCCGCATCCGCTCACGCTCCC |
| | reverse primer: | CCATCGATGGCTGGCAGTTCGCACCCTA |

**3. Primers for *GPAM* promoter site-directed mutagenesis**

| | |
|---|---|
| (-428/+86) GPAM mutation forward | GTGAGCGCCTGGACGGACGGTTACCCTGC CTA |
| (-428/+86) GPAM mutation reverse | TAGGCAGGGTAACCGTCCGTCCAGGCGCTC AC |

**4. Primers used for *BMAL1* site-directed mutagenesis**

| | |
|---|---|
| BMAL1 mutation forward | GTATTATAGAACAAGGCTTCTCTTTATTAAA AATATTTAACTGTCTAGT |
| BMAL1 mutation reverse | ACTAGACAGTTAAATATTTTTAATAAAGAG AAGCCTTGTTCTATAATAC |

**5. Primers used for ChIP in the *GPAM* promoter**

| | | |
|---|---|---|
| *GPAM* | forward primer | GCAACAGATGAATCCCTA |
| | reverse primer | TGAATTTCCTGAGAACCC |

**6. siRNAs**

| Gene | sense | anti-sense |
|---|---|---|
| siBMAL1-1 | GCUCUUUCUUCUGUAGA AUTT | AUUCUACAGAAGAAAGAGCTT |
| siBMAL1-2 | GCCUUCAGUAAAGGUUG AA | UUCAACCUUUACUGAAGGC |
| siGPAM | CAAUCAAAAGCCGUUAA CA | UGUUAACGGCUUUUGAUUG |
| siCLOCK-1 | ACGAGAACTTGGCATT GAA | UUCAAUGCCAAGUUCUCGU |
| siCLOCK-2 | CAAGATTCTGGGTCAG ATA | UAUCUGACCCAGAAUCUUG |
| siControl | UUCUCCGAACGUGUCAC GUTT | ACGUGACACGUUCGGAGAATT |

**7. miR-494-3p and anti-mi-494-3p**

| Gene | sense | anti-sense |
|---|---|---|
| miR-494-3p mimics | UGAAACAUACACGGGAAA CCUC | GGUUUCCCGUGUAUGUUUCAUU |
| miR-494-3p inhibitor | GAGGUUUCCCGUGUAUGU UUCA | |
| miR-27b-3p mimics | UUCACAGUGGCUAAGUUC UGC | AGAACUUAGCCACUGUGAAUU |
| miR-141-3p mimics | UAACACUGUCUGGUAAAG AUGG | ACAUAGGAAUAAAAAGCCAUAU U |
| miR-141-3p mimics | UAACACUGUCUGGUAAAG AUGG | AUCUUUACCAGACAGUGUUAUU |
| miR-155-5p mimics | UUAAUGCUAAUCGUGAUA GGGGUU | CCCCUAUCACGAUUAGCAUUAAU U |

**Table S2:** Primary antibodies used in this study.

| Antibody | Company (Cat. No.) | Working dilutions |
|---|---|---|
| BMAL1 | NOVUS (NB100-2288) | WB: 1/1000; IHC:1/1000; IF: 1/200; IP:1/100; ChIP:1/100; |
| GPAM | SANTA (sc-398135) | WB: 1/1000; IHC:1/200 |
| EZH2 | CST (3147S) | IF: 1/200 |
| EZH2 | Abcam (191250) | IP:1/50 |
| cleaved caspase-9 | Abcam(ab2324) | WB: 1/1000 |
| cleaved caspase-3 | Abcam(ab2302) | WB: 1/500 |
| cleaved PARP | Abcam(ab194217) | WB: 1/1000 |
| CDK4 | Proteintech(11026-1-AP) | WB: 1/500 |
| CDK6 | Proteintech (14052-1-AP) | WB: 1/1000 |
| Cyclin D1 | Proteintech(60186-1-AP) | WB: 1/1000 |
| E-cadherin | Proteintech(20874-1-AP) | WB: 1/500 |
| ZO-1 | Proteintech(21773-1-AP) | WB: 1/1000 |
| N-cadherin | Cell Signaling (13116) | WB: 1/1000 |
| Vimentin | Proteintech(10366-1-AP) | WB: 1/1000 |
| Ki-67 | Abcam(ab15580) | IHC:1/250 |
| CLOCK | Proteintech(13463-1-AP) | WB:1/500 |
| β-actin | Beijing TDY(TDY051F) | WB: 1/3000 |

**Table S3: Correlations between expressions of BMAL1 and clinic-pathologic features of 217 HCC patients.**

| Variables | No. of cases (%) | BMAL1 expression Low | BMAL1 expression High | *P* value |
|---|---|---|---|---|
| All | 217 (100%) | 118 | 99 | |
| Age | | | | |
| <55 | 53 | 25 | 28 | 0.226 |
| >=55 | 164 | 93 | 71 | |
| Gender | | | | |
| Female | 35 | 19 | 16 | 0.991 |
| Male | 182 | 99 | 83 | |
| HBV | | | | |
| Negative | 15 | 7 | 8 | 0.557 |
| Positive | 202 | 111 | 91 | |
| AFP (ug/ml) | | | | |
| <200 | 159 | 86 | 73 | 0.887 |
| >=200 | 58 | 32 | 26 | |
| Maximum diameter of lesion | | | | |
| <5 | 92 | 42 | 50 | **0.027** |
| >=5 | 125 | 76 | 49 | |
| PVTT | | | | |
| No | 191 | 97 | 94 | **0.004** |
| Yes | 26 | 21 | 5 | |
| TNM stage | | | | |
| I+ II | 32 | 22 | 10 | 0.078 |
| III+ IV | 185 | 96 | 89 | |
| Treatment | | | | |
| Hepatectomy | 90 | 48 | 42 | 0.795 |
| Hepatectomy+ TACE | 127 | 70 | 57 | |

**Abbreviations**: AFP, alpha-fetoprotein; PVTT, portal vein tumor thrombosis; TNM, tumor-nodes-metastases; TACE, transcatheter arterial chemoembolization.

**Table S4: Univariate analysis of potential predictors for OS in 217 HCC patients.**

| Variables | No. of cases (%) | Median of OS time(months) | *P* value |
|---|---|---|---|
| Age | | | |
| <55 | 53 | 33.1 | 0.829 |
| >=55 | 164 | 37.8 | |
| Gender | | | |
| Female | 35 | 33.5 | 0.105 |
| Male | 182 | 39.5 | |
| HBV | | | |
| Negative | 15 | 41.3 | 0.495 |
| Positive | 202 | 34.7 | |
| AFP (ug/ml) | | | |
| <200 | 159 | 40.3 | **0.001** |
| >=200 | 58 | 11.6 | |
| Maximum diameter of lesion | | | |
| <5 | 92 | 40.5 | **0.011** |
| >=5 | 125 | 30.4 | |
| PVTT | | | |
| No | 191 | 39.9 | **0.001** |
| Yes | 26 | 11.6 | |
| TNM stage | | | |
| I+ II | 32 | 46.6 | **0.003** |
| III+ IV | 185 | 31.8 | |
| Treatment | | | |
| Hepatectomy | 90 | 34.9 | 0.375 |
| Hepatectomy+ TACE | 127 | 37.8 | |

**Table S5: Univariate analysis of potential predictors for RFS in 217 HCC patients.**

| Variables | No. of cases (%) | Median of RFS time (months) | *P* value |
|---|---|---|---|
| Age | | | |
| <55 | 53 | 8.3 | 0.413 |
| >=55 | 164 | 11.0 | |
| Gender | | | |
| Female | 35 | 14.8 | 0.410 |
| Male | 182 | 8.5 | |
| HBV | | | |
| Negative | 15 | 9.0 | 0.631 |
| Positive | 202 | 18.3 | |
| AFP (ug/ml) | | | |
| <200 | 159 | 17.4 | **0.001** |
| >=200 | 58 | 2.6 | |
| Maximum diameter of lesion | | | |
| <5 | 92 | 17.7 | **0.001** |
| >=5 | 125 | 6.7 | |
| PVTT | | | |
| No | 191 | 15.0 | **0.001** |
| Yes | 26 | 2.5 | |
| TNM stage | | | |
| I+ II | 32 | 24.6 | 0.001 |
| III+ IV | 185 | 7.3 | |
| Treatment | | | |
| Hepatectomy | 90 | 9.3 | 0.239 |
| Hepatectomy+ TACE | 127 | 11.0 | |

**Table S6. Multivariate analysis of potential predictors for OS in 217 HCC patients（BMAL1）.**

| Variables | HR | 95%CI | *P* value |
|---|---|---|---|
| AFP | 2.453 | 1.676-3.590 | 0.001 |
| Maximum diameter of lesion | 1.785 | 1.201-2.653 | 0.004 |
| PVTT | 3.678 | 2.208-6.128 | 0.001 |
| TNM stage | 1.877 | 1.007-3.499 | 0.047 |
| BMAL1 | 0.598 | 0.403-0.889 | 0.011 |

**Table S7. Multivariate analysis of potential predictors for RFS in 217 HCC patients (BMAL1).**

| Variables | HR | 95%CI | *P* value |
|---|---|---|---|
| AFP | 2.404 | 1.700-3.401 | 0.001 |
| Maximum diameter of lesion | 1.849 | 1.288-2.654 | 0.001 |
| PVTT | 2.930 | 1.788-4.802 | 0.001 |
| TNM stage | 2.610 | 1.424-4.784 | 0.002 |
| BMAL1 | 0.511 | 0.358-0.731 | 0.001 |

**Table S8. Multivariate analysis of potential predictors for OS in 217 HCC patients（GPAM）.**

| Variables | HR | 95%CI | *P* value |
|---|---|---|---|
| AFP | 2.649 | 1.811-3.876 | 0.001 |
| Maximum diameter of lesion | 2.023 | 1.364-3.001 | 0.001 |
| PVTT | 5.119 | 3.112-8.420 | 0.001 |
| GPAM | 2.257 | 1.494-3.410 | 0.001 |

**Table S9. Multivariate analysis of potential predictors for RFS in 217 HCC patients (GPAM).**

| Variables | HR | 95%CI | *P* value |
|---|---|---|---|
| AFP | 2.437 | 1.720-3.453 | 0.001 |
| Maximum diameter of lesion | 2.097 | 1.469-2.996 | 0.001 |
| PVTT | 4.079 | 2.508-6.636 | 0.001 |
| TNM stage | 1.988 | 1.088-3.633 | 0.026 |
| GPAM | 2.057 | 1.412-2.997 | 0.001 |

## Reference


1. Yuan P, Li J, Zhou F, Huang Q, Zhang J, Guo X, et al. NPAS2 promotes cell survival of hepatocellular carcinoma by transactivating CDC25A. Cell death & disease. 2017; 8: e2704.
2. Zhao J, Zhang X, Gao T, Wang S, Hou Y, Yuan P, et al. SIK2 enhances synthesis of fatty acid and cholesterol in ovarian cancer cells and tumor growth through PI3K/Akt signaling pathway. Cell Death Dis. 2020; 11: 25.
3. Zhao J, Hou Y, Yin C, Hu J, Gao T, Huang X, et al. Upregulation of histamine receptor H1 promotes tumor progression and contributes to poor prognosis in hepatocellular carcinoma. Oncogene. 2020; 39: 1724-38.
4. Zhang H, Fan J, Wang J, Dou B, Zhou F, Cao J, et al. Fluorescence discrimination of cancer from inflammation by molecular response to COX-2 enzymes. J Am Chem Soc. 2013; 135: 17469-75.
5. Zhang H, Fan J, Wang K, Li J, Wang C, Nie Y, et al. Highly sensitive naphthalene-based two-photon fluorescent probe for in situ real-time bioimaging of ultratrace cyclooxygenase-2 in living biosystems. Anal Chem. 2014; 86: 9131-8.
